# Characterizing patterns of seasonal drought stress for use in common bean breeding in East Africa under present and future climates

**DOI:** 10.1016/j.agrformet.2023.109735

**Published:** 2023-11-15

**Authors:** Prakash K. Jha, Steve Beebe, Patricia Alvarez-Toro, Clare Mukankusi, Julian Ramirez-Villegas

**Affiliations:** a International Center for Tropical Agriculture (CIAT), Km 17, Recta Cali-Palmira, Cali, Colombia; b Division of Agriculture and Natural Resources, University of California, Merced, CA, United States; c Bioversity International, Via di San Domenico, 1, 00153, Rome, Italy; d CGIAR Research Program on Climate Change, Agriculture and Food Security (CCAFS), c/o CIAT, Palmira, Colombia; e Plant Production Systems Group, Wageningen University and Research, Wageningen, the Netherlands; f International Center for Tropical Agriculture (CIAT), P. O. Box 6247, Kampala, Uganda

**Keywords:** Target population of environments, DSSAT, Water-stress, Climate change, Adaptation, Crop improvement

## Abstract

Common bean (*Phaseolus vulgaris* L.) is the second most important source of dietary protein and the third most important source of calories in Africa, especially for the poor. In East Africa, drought is an important constraint to bean production. Therefore, breeding programs in East Africa have been trying to develop drought resistant varieties of common bean. To do this, breeders need information about seasonal drought stress patterns including their onset, intensity, and duration in the target area of the breeding program, so that they can mimic this pattern during field trials. Using the Decision Support for Agrotechnology Transfer (DSSAT) v4.7 model together with historical and future (Coupled Model Inter-comparison Project 6, CMIP6) climate data, this study categorized Ethiopia, Tanzania, and Uganda into different target population of environments (TPEs) based on historical and future seasonal drought stress patterns. We find that stress-free conditions generally dominate across the three countries under historical conditions (50–80% frequency). These conditions are projected to increase in frequency in Ethiopia by 2–10% but the converse is true for Tanzania (2–8% reduction) and Uganda (17–20% reduction) by 2050 depending on the Shared Socioeconomic Pathway (SSP). Accordingly, by 2050, terminal drought stresses of various intensities (moderate, severe, extreme) are prevalent in 34% of Uganda, around a quarter of Ethiopia, and 40% of the bean growing environments in Tanzania. The TPEs identified in each country serve as a basis for prioritizing breeding activities in national programs. However, to optimize resource use in international breeding programs to develop genotypes that are resilient to future projected stress patterns, we argue that common bean breeding programs should focus primarily on identifying genotypes with tolerance to severe terminal drought, with co-benefits in relation to adaptation to moderate and extreme terminal drought. Little to no emphasis on heat stress is warranted by 2050s.

## Highlights

• Bean growing areas in EAF experience six drought stress patterns across the seasons.
• The drought stress conditions are likely to increase in frequency in future.
• The drought stress conditions hardly occur with heat stress.
• The drought stress characterization can guide bean breeding.

## 1 Introduction

Common bean (Phaseolus vulgaris L.) has important dietary and nutritional values especially for the poor in Africa (Beebe et al., 2011; Gardner and Kassebaum, 2020; Hummel et al., 2018; Izquierdo et al., 2018; Pachico, 1993). The demand for bean is expected to grow further during the 21st century, as the proportion of nutrient rich legumes in East African diets increases with the rise in income and urbanization (Beebe, 2020; Gouel and Guimbard, 2019; Katungi et al., 2009). In Tanzania and Uganda, a recent *ex-ante* analysis suggests projected increases in bean demand of 157% and 203% (respectively) (Schiek et al., 2021).

Whereas demand for common bean is projected to increase across Eastern Africa (EAF, hereafter), several studies project reductions in the supply of bean due to climate change (Beebe et al., 2011; Hummel et al., 2018; Taba-Morales et al., 2020). Drought, waterlogging, heat stress, and low phosphorous are the most important abiotic stresses that limit bean productivity across EAF (Araújo et al., 2015; Assefa et al., 2019; Beebe, 2012). Mediated by reductions in climatically suitable area for beans due to temperature increases, these studies suggest substantial reductions in bean growing areas by 2030 and 2050 unless adaptation actions are pursued (Beebe et al., 2011; Taba-Morales et al., 2020). Temperatures also drive crop yield variations, as common beans are reportedly sensitive to high temperatures during the night (Deva et al., 2020; Porch et al., 2010). Model-based studies show that in low elevation areas where temperatures are relatively high and crops often experience drought stress, climate change causes productivity decreases (Thornton et al., 2009). In contrast, highland areas have been shown to increase yield under climate change (Mourice et al., 2016).

The breeding of new varieties with higher yield potential and the ability to withstand stress conditions, will likely be the cornerstone of bean adaptation to climate change (Araújo et al., 2015; Beebe et al., 2011). But the focus of plant breeding for climate adaptation is likely to change depending on how different abiotic stresses evolve and act during the growing season along with their relative importance. For example, Heinemann et al. (2017) suggested that the Brazilian bean breeding program should increase their emphasis on bean varieties with drought stress tolerance. Likewise, Ramirez-Villegas et al. (2018) recommended modifying the selection strategy for rice breeding based on the predicted stresses over the target area of the breeding program under climate change. To the best of our knowledge, however, there are no studies that analyze implications of shift in drought stress for bean breeding in Africa.

In this study, we investigate the current and future changes in frequency, duration and intensity of drought stress resulting from climate change for rainfed bean production in the context of three east African countries (Ethiopia, Uganda, and Tanzania). Using crop and climate models, we analyzed whether and how future projected increases in temperature and atmospheric CO_2_ concentrations, and changes in precipitation are likely to alter the seasonal drought stress characteristics of bean growing areas in the three countries. We also analyzed the variation in average minimum temperatures, to understand the co-occurrence of thermal stress environments with drought stress. Based on these analyses, we discuss implications for bean breeding practice for East Africa in the coming decades.

## 2 Materials and methods

### 2.1 Overview

We conducted bean crop growth and development simulations over all bean growing areas of Ethiopia, Uganda, and Tanzania using the CROPGRO-DRYBEAN crop simulation model of the Decision Support System for Agrotechnology Transfer (DSSAT) version 4.7 (DSSAT v4.7, hereafter, see Sect. 2.4 and 2.6 for details) (Hoogenboom et al., 2019a, 2019b). We ran simulations under 1991–2010, hereafter referred as the ‘historical’ climate, 2021–2040, hereafter referred as the ‘near-future’ climate and 2041–2060, hereafter referred as the ‘far-future’ climate. We used four Shared Socioeconomic Pathways (SSP1–2.6, SSP2–4.5, SSP3–7.0 and SSP5–8.5), ranging from lowest to highest in terms of GHG emission, and five General Circulation Models (GCMs) (see Sect. 2.3 for details). We then took the simulation output water stress (WSPD –water stress index for photosynthesis) and average minimum temperature data to classify bean areas first into a set of environments (see Sect. 2.7). Finally, we analyzed these environments across bean production hubs (see Sect. 2.2) and discuss implications for bean breeding.

### 2.2 Bean production hubs

**Fig. 1:**
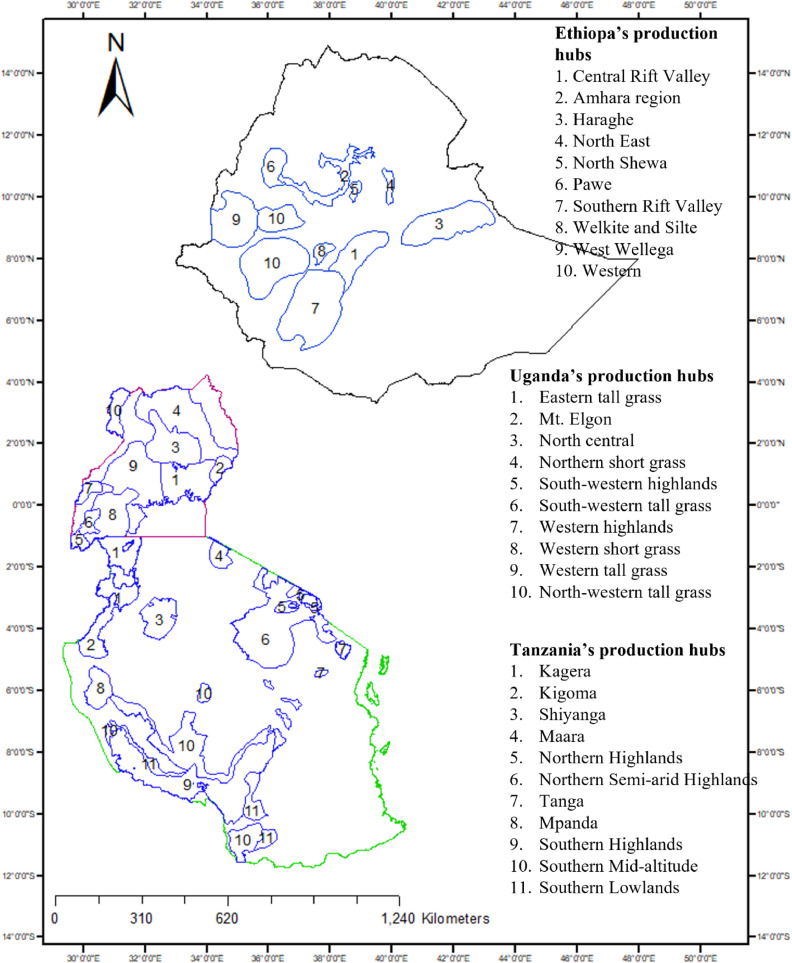
Maps of Ethiopia (black border), Uganda (purple border) and Tanzania (green border) along with a list of the production hubs in each country (blue border).

The crop improvement program targets ‘bean corridors’ in EAF, which are areas of (economic and production) intensification characterized by flows of beans from production to consumption. Within a bean corridor, the production hubs represent high production potential areas for bean that share certain biophysical and socioeconomic characteristics, serve specific market segments in EAF, and involve large numbers of smallholder and other farmers. The common bean improvement programs focus their efforts on developing genotypes that have the market characteristics preferred in each production hub and can tolerate the biotic and abiotic stresses empirically known to exist in them (Fig. 1). Further details on these production hubs including their biophysical and socioeconomic characteristics are reported in the Bean Atlas (Farrow and Muthoni-Andriatsitohaina, 2020).

### 2.3 Weather and soil data

We used historical observations from 1991 to 2010 of daily maximum temperature (*T_max_*), minimum temperature (*T_min_*), solar radiation (*S_rad_*) from the AgMERRA gridded dataset (Ruane et al., 2015) available at a resolution of 0.25°x0.25° along with precipitation from the Climate Hazards Infra-Red Precipitation with Stations dataset (CHIRPS, Funk et al., 2015), available at a resolution of 0.05°x0.05° Future climate projections of these climate variables were obtained from an ensemble of five GCMs and four different SSPs. Specifically, we derived these climate projections from General Circulation Models (GCMs) participating in the Coupled Model Inter-comparison Project 6 (CMIP6, Eyring et al., 2016). Since the concentration of GHG in the future depends on evolution of various socioeconomic and technological factors, there are various pathways, called Shared Socioeconomic Pathways (SSP), to describe the future scenarios of greenhouse gas emissions (GHG) (Meinshausen et al., 2020). We used four Shared Socioeconomic Pathways (SSP1–2.6, SSP2–4.5, SSP3–7.0 and SSP5–8.5), ranging from lowest to highest in terms of GHG emission. For each SSP, simulations exist for many GCMs. Here, we use five GCMs — BCC-CSM2-MR, EC-Earth3-Veg, GFDL-ESM4, IPSL-CM6A-LR and MRI-ESM2–0. The GCMs and SSPs used in this study were chosen to represent the maximum possible spread in future climate and to have complete data of the four weather variables for four SSP.

The combination of five GCMs, four SSPs and two periods (‘near future’ and ‘far future’) gives 40 different climate scenarios for our simulation analysis. The CMIP6 future climate projections data had spatial resolutions ranging from 0.75°x0.75° to 2.5°x2.5° and were available at monthly frequency. Since data from GCMs cannot be used directly as input into crop model simulations due to their spatial scale and accuracy (Hansen et al., 2006; Ramirez-Villegas et al., 2013; Semenov and Porter, 1995), the future climate data were bias corrected. For maximum and minimum temperatures, we followed the Change Factor (CF) method of Hawkins et al. (2013) considering changes in both mean and variance in the bias correction. Precipitation and solar radiation were bias corrected following Navarro-Racines et al. (2020), who apply the delta method (DM) using relative changes to the monthly means. This was done since the CF method is not suitable for precipitation and solar radiation (which are accumulated values over a day) and could result in negative values, which would be unrealistic. Since the output of the bias correction is at the spatial and temporal resolution of the historical data (see Hawkins et al., 2013), this means that bias corrected weather data for future climate projections were at a spatial resolution of 0.05°x0.05° and at a daily frequency for input into the crop model. Soil data were obtained from a global soils profile database for crop modeling applications (IRI et al., 2015). This database was created on the basis of the International Soil Reference and Information Center (ISRIC) SoilGrids database, at a resolution of 5 arc-min, and includes all the variables required for DSSAT v4.7 model simulations, namely, soil hydrological parameters, bulk density, texture, nitrogen content, pH in water, Cation exchange capacity, and organic carbon content percentage at various depths (Hengl et al., 2014). For Uganda, Ethiopia and Tanzania, there were in total 2545, 9706 and 10,647 unique soil profiles respectively, and depth-specific soil properties of these soil profiles are available in IRI et al. (2015).

### 2.4 Crop model

We ran simulations of common bean growth and development using the CROPGRO-DRYBEAN model included within the DSSAT v4.7 Cropping Systems Model (CSM). CROPGRO-DRYBEAN is a process-based model that runs on a daily timestep using modules for crop development, carbon balance, water balance, and nitrogen balance. We choose the CROPGRO model because we are interested in understanding water stress experienced by common bean plants resulting from the interactions of increase in temperature, change in precipitation, and increase in atmospheric CO_2_ concentration based on physiological processes including their impact on photosynthesis, partitioning, root water uptake, and actual transpiration from plants. CROPGRO takes these factors into account in a mechanistic fashion. Moreover, the model has also been tested extensively (Boote et al., 1998, 2018), including under water-limited conditions (Heinemann et al., 2016; Heinemann et al., 2017), and has been used previously in Africa to simulate bean yield under future climate (Thornton et al., 2009).

The species file of CROPGRO-DRYBEAN model includes temperature thresholds to describe plant development rate at various development stages, coefficients to limit photosynthesis according to daily maximum and minimum temperatures and accounts change in photosynthesis with the increase in CO_2_ concentration (Boote et al., 1998). The plant water stress index related to photosynthesis (WSPD) is simulated by DSSAT v4.7 on a daily basis based on the ratio of actual to potential transpiration, and is used to reduce photosynthesis when there is water stress. The water balance module of the model uses a one-dimensional multi-layer tipping bucket approach, (Ritchie, 1998). This approach is well established in the crop modeling community, and produces realistic water balance simulations and soil moisture predictions in a wide range of conditions (Boote et al., 2008, 2018; Ritchie et al., 2009). In the water balance, the model simulates potential evapotranspiration (PET) using the Priestley-Taylor equation (Priestley and Taylor, 1972). The model-simulated Leaf Area Index (LAI) and the energy extinction coefficient are used to split potential PET into soil water evaporation (PE) and plant transpiration (PT). Then, the model calculates the soil evaporation based on the actual soil water contents. The actual evaporation is the minimum of PE and the calculated soil evaporation value. Whether or not plants will get enough water to meet their PT demand depends on the root length density in each soil layer, the rate of water uptake per unit of root length, and the extractable soil water content and depth of each soil layer. The root length density is partitioned based on biomass and stages of development. The extractable soil water for each layer is calculated by subtracting the soil water from the drained lower limit of the soil. The actual plant transpiration is the minimum of PT and the value obtained from root water uptake calculation. Further details on soil water balance are available in Ritchie (1998) and Boote et al. (2008, 2018).

### 2.5 Model calibration and evaluation

Our simulations required specifying a cultivar. We used cv. Calima, a commonly cultivated bush bean variety in eastern and southern African countries, which represents the determinate growth habit within the Andean gene pool. Calima has also been used in previous modeling studies as a representative cultivar for EAF (Ramirez-Villegas and Thornton, 2015; Thornton et al., 2009). In all countries, simulations used cv. Calima as it represents many varieties used throughout the region, but also because using the same cultivar allowed comparing results across countries. To be able to confidently use the cv. Calima in DSSAT v4.7 for the TPE analysis, the first step was to calibrate and evaluate the model using field trial data. Calibration involves determining the model parameters (also referred to as genotype-specific parameters, GSPs). Cultivar parameters define the phenology, rates of growth of specific organs, as well as how the assimilated carbohydrates are distributed in different parts of plants.

We used data from bean experimental trials conducted in 2013 by the CIAT bean program in Villanueva Municipality in the department of Santander (Colombia), a highland locality highly representative of East African bean growing environments according to the climate similarity analysis (Ramírez-Villegas et al., 2011; Aggarwal et al., 2018), since no experiments were available in East Africa that would allow detailed model calibration and evaluation for cv. Calima. There were six different trials sown on six different planting dates: 19th April 2013 (harvested 26th June, corresponding to season 1 –P1), 6th September of 2013 (harvested 11th December 2013, season 2 –P2), 8th January 2014 (harvested 26th March, corresponding to season 3 – P3), 14th April 2014 (harvested 30th June – 5th July, corresponding to season 4 – P4), 10th July 2014 (harvested 25th September – 3rd October, corresponding to season 5 – P5), and 18th September 2014 (harvested 19th December, corresponding to season 6 – P6). Genotypes were planted in a randomized complete block design with three replications and six treatments. Two treatments were used for calibration (P1 and P2), and another two for evaluation (P3 and P5). Plants were randomly sampled every 15 days from the sowing date to measure and record phenology and growth variables. The phenological stages were identified following the criteria from Fernández et al. (1986). Growth variables recorded included leaf area (cm^2^), foliar biomass (g), stem biomass (g), flower and pod biomass (g), foliar dry weight (g), stem dry weight (g), and flower and pod dry weight (g). These data were included into corresponding “T” and “A” files. For all treatments, three fertilizers, namely, Calcium Nitrate containing Nitrogen 4 kg ha^−1^, Ammonium Sulfate containing 16 kg ha^−1^ of Nitrogen and Single Super Phosphate containing 26 kg ha^−1^ of Phosphorus, were incorporated at a depth of 5 cm on the day of sowing. Fungicides were applied in all treatments.

The calibration process included two steps: (1) sensitivity analysis to select the most relevant model parameters using Sobol’ et al. (2007)’s methodology to partition the variance from each parameter, and (2) parameter calibration, using genetic algorithms (Guo et al., 2021; Jones et al., 2011; Li et al., 2018), aimed at obtaining a set of cultivar parameters that gives the lowest root mean square error (RMSE) between the measured data (yield, total biomass, LAI, days to flowering and days to maturity) from field trials and the mode-simulated outputs. Details on climate similarity analysis, sensitivity analysis and parameter calibration processes are available in Supplementary Text S1.

Water and nitrogen limited bean growth and yield were simulated for the Villanueva experimental site using DSSAT v4.7 simulation model. The inputs needed to run the DSSAT v4.7 are weather, soil, agronomic management, and cultivar genetic coefficients. Daily precipitation was recorded on site using a pluviometer, whereas maximum and minimum temperatures were estimated using nearby weather stations following Gourdji et al. (2013). Solar radiation was estimated directly by DSSAT v4.7. Depth-specific soil texture, soil-hydrological parameters were measured along with bulk density and pH, and a soil profile “Laja Villanueva” (ccCCLA1310) was added in the SOIL.SOL file using these measured data. The default model parameters of cv. Calima in DSSAT v4.7, available from (Clavijo Michelangeli et al., 2014, 2013) were used as an initial value for the calibration process.

Using the calibration process, we obtained the final set of cultivar parameters (Table S1), using which the model was able to capture the dynamic progress in Leaf Area Index (LAI), weight of dry leaves (LWAD), above-ground dry weight of plants (CWAD), stem dry weight (SWAD) and dry weight of grain (GWAD) over the growing season (Figure S2). The model was able to reproduce phenology (anthesis day, first pod day, first seed day and physiological maturity day) of the first trial with an error ranging from 0 to 5 days but for the second trial, the difference between model simulated and observed physiological maturity days was 14 days (Table S2). The same set of cultivar parameters was used to evaluate model performance for the same site but for third and fifth sowing dates in 2014. Again, the model correctly captured the progress in LAI, LWAD, CWAD and SWAD over the growing season (Figure S3). The model simulated phenology with an error ranging from 1 to 5 days compared to the observed phenology for the third sowing dates but for the fifth sowing date, the model simulated physiological maturity was 17 days late than the observed value (Table S3). Further evidence of the ability of the CROPGRO-DRYBEAN model to simulate cv. Calima performance exists in recent literature. Most notably, Alvarez et al. (2020) carried out an evaluation of the model for cv. Calima using experimental data from bean trial established in 2019 and 2020 at the Altobonito farm, Vereda los Tendidos, Municipality of Popayán in the department of Cauca, located at latitude 2° 29′ 24′' N, longitude 76° 39′ 45′' W, at an altitude of 1742 m.a.s.l. (representative of many highland locations in East Africa). Using the cultivar parameters of Table S1, the model showed an error of up to 3 days in simulating number of days after sowing needed for anthesis, first pod and physiological maturity, with larger errors being observed for the day of first seed appearance (Table S4). Also, the model performed very well in reproducing CWAD, LWAD, pod dry weight (PWAD). However, the model somewhat diverged from observations for LAI, and SWAD. Regarding yield, the model simulated yield (2032 kg ha-1) was only 6.5% above the measured yield (1907 kg ha^−1^). Despite some limitations, we argue that the model captures the dynamics of rainfed bean production, and hence can be used for environmental characterization.

### 2.6 Crop model simulations for the three countries

Using the cultivar parameters from the calibration exercise, water-limited bean productivity was simulated for these three countries using DSSAT v4.7 for present and future climate for every grid point at a resolution of 0.05°x0.05° Weather and soil inputs were as in Sect. 2.3. For each country, historical simulations used observed weather data, whereas future simulations were conducted for the 40 individual future climate projections. Atmospheric CO_2_ concentration input into the crop model for the SSPs 1–2.6, 2–4.5, 3–7.0, and 5–8.5 for the 2030 (2050) periods were 440 (469), 446 (508), 454 (544), and 456 (569) parts per million respectively (Meinshausen et al., 2020).

Sowing dates were defined based on the Food and Agricultural Organization (FAO) of the United Nations calendar (FAO, 2021), National Agricultural Research System (NARS) data, the Bean Atlas (Farrow and Muthoni-Andriatsitohaina, 2020), and direct consultation with the International Center for Tropical Agriculture (CIAT) African crop improvement team. In the case of Uganda, we found that bean is cultivated continually starting from mid-February until December. At an interval of seven days, this resulted in 34 sowing dates for the entire Feb-Dec period. When clustering the water stress patterns and temperatures (see Sect. 2.7), results from each sowing date were given equal weight. Tanzania has two growing seasons for bean, although some regions have just one. We sampled four sowing dates in each season at an interval of a week, for a total of eight sowing dates. In Ethiopia, bean is sown mainly from early July to Mid-September, depending on the region, although there are some regions in eastern Ethiopia where bean is sown in early March as well. This resulted in three to nine sowing dates, at an interval of seven days, depending on the region. Following discussions with bean crop improvement and agronomy experts across East Africa, all planting dates were given the same weight. Since this study is concerned with water stress and its impact, we assumed optimum nitrogen supply. Considering all climate scenarios, management options and locations, a total of ca. 655 million simulations were performed.

### 2.7 Seasonal drought stress pattern and temperature classification

We conducted the drought stress analysis for each country separately since we seek to produce results that can best help tailor the crop improvement strategy for each country and within each country for each bean production hubs. Similarly, we analyzed climate scenarios separately since each future climate scenario is considered equally plausible, and analyzing each one individually helps explore the range of outcomes that could be expected and their implications for crop improvement.

We categorized each country into target population of environments (TPEs) based on the seasonal variation of the simulated water stress (WSPD). We used the WSPD because it is directly linked to carbon fixation and therefore total biomass accumulation, which means we can verify the model's robustness in representing this process against total biomass measurements. This increases confidence in our results. Furthermore, the relationship between photosynthesis and water dynamics in the soil and plant (through soil desiccation, root water uptake and stomatal conductance) are also well represented in the model (see Boote et al., 1998, Boote et al., 2018). For each growing season, we first calculated the average WSPD, for each of the three vegetative (V1, V2, V3) and five reproductive growth stages (R1, R3, R5, R7 and R8) based on the criteria of Fehr et al. (1971) using the entire set of simulations (i.e., all planting dates x 20 years). According to Fehr et al. (1971) common bean plants with less than two leaves were categorized into V1, two to five leaves into V2 and more than five leaves (until flowering) were categorized into V3 stage. Similarly, common bean plants are considered in R1, R3 and R5 stages if half of the plants have at least one flower, 0.5 cm pod and bean seeds beginning to develop, respectively. R7 is the physiological maturity when pods start yellowing and R8 is the harvest maturity. For each country and climate scenario individually, we therefore compiled a matrix containing all grid cell locations, years, and planting dates (as rows), and with WSPD values averaged for the eight growth stages (V1–V3, R1, R3, R5, R7 and R8) as columns.

To create the TPEs, we used Ward's agglomerative hierarchical clustering with the incremental sum of squares as the criteria for deciding number of environmental groups (Ward, 1963). For the historical period, the number of environmental groups was defined through the within-group sum of squares, and breeders’ knowledge of the bean growing environments in Eastern Africa. The breeders’ knowledge was used primarily to check whether the geographic area corresponding to each TPE was consistent with the anecdotal knowledge of the breeders. Corrections in simulation design (e.g., planting dates) were made as needed following these checks. The number of environmental groups in the historical period was kept constant for the future scenarios. Clustering analyses were performed using the *fastcluster* package in the R statistical framework (Müllner, 2018).

Depending on the sowing date and year, the same grid point can be under different environmental groups. From these we calculated the most frequent environmental group for each grid for each country, model, and SSP, and created maps of the most frequent TPE. For each TPE, we summarized the seasonal WSPD behavior using the median and the interquartile range. To visually inspect the differences in onset, duration, and intensity of water stress between TPEs across the climate scenarios, we produced plots of seasonal TPE stress patterns using the 25th and 75th percentiles of water stress and smoothened the lines using a loess regression (Cleveland et al., 2017). We calculated bean harvested area under each TPE in each country by multiplying the total bean harvested area data (spam2017v2r1_SSA_H_BEAN_A.tif) from MapSPAM (IFPRI, 2020) in each country with the proportion of each TPE in each country. Proportion of each TPE in each country was computed based on most frequent TPE in each grid out of the total number of grids in each country. To make information from this research more tailored to the crop improvement program, we pooled and presented results according to existing production hubs for this purpose.

We performed the temperature analysis separately. Toward this aim, we defined thermal environments (TEs) through fixed classes of average seasonal minimum temperature. We use minimum temperatures because the most experimental evidence suggests that high nighttime temperature reduce bean yield (Beebe et al., 2011; Rainey and Griffiths, 2005; Prasad et al., 2002; Deva et al., 2020). Thus, we use minimum temperature as a proxy for nighttime temperature. The classes were defined jointly with the crop improvement team based on thresholds of heat stress for common bean (also see Beebe et al., 2011). The classes were (1) <15 °C; (2) 15–18 °C; (3) 18–20 °C; (4) 20–22 °C; and (5) 22–25 °C. Finally, we quantified the amount of bean harvested area under each temperature class including the area under heat stress (T_min_ > 22 °C) and its overlap with the drought stress patterns.

## 3 Results

### 3.1 Projected changes in bean yield

**Fig. 2:**
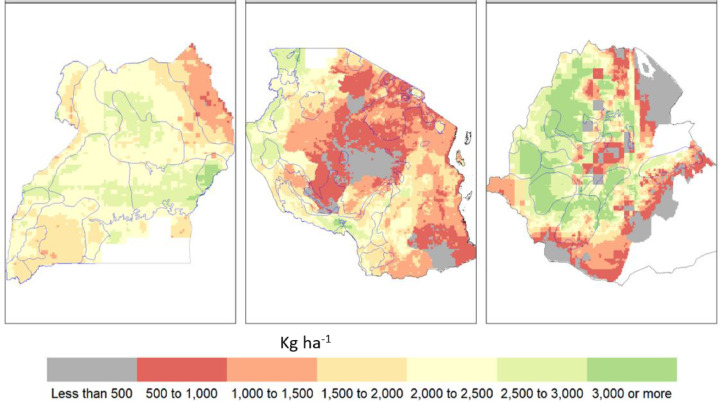
Simulated bean yield in the historical period 1991–2010 for Uganda (left), Tanzania (middle) and Ethiopia (right). The thin blue polygons represent different bean corridors.

**Fig. 3:**
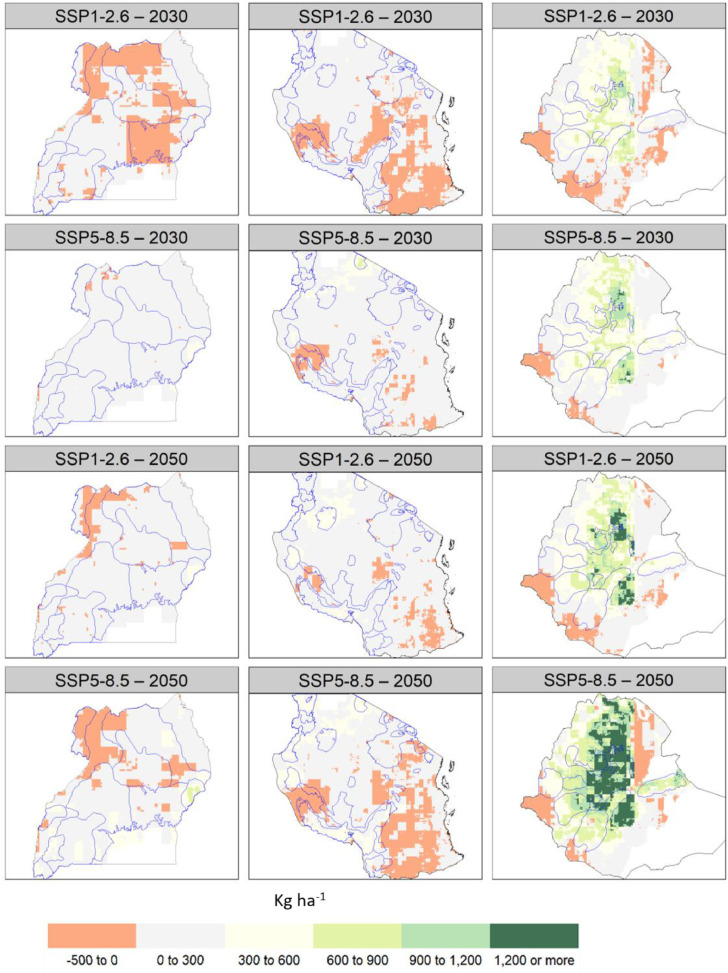
Projected change in bean yield in 2021–2040 (the upper two rows) and in 2041–2060 (the bottom two rows) compared to the historical period 1991–2010 for Uganda (left), Tanzania (middle) and Ethiopia (right). The thin blue polygons represent different bean production hubs.

Across the three countries studied, bean crop yield is projected to change (positively or negatively), sometimes significantly, from its historical mean (Fig. 2, Fig. 3, and Figure S4 for other SSPs). In the vast majority of bean growing areas, crop-climate model projections suggest moderate increases in yield (<300 kg ha^−1^), and only in Ethiopia yield increases are more substantial (more than 1200 kg ha^−1^). In some of these areas, simulated historical yield is low (<1000 kg ha^−1^), implying that many of these regions increase significantly their potential for bean cultivation. This is likely because of greater carbon fixation from increased CO_2_ concentrations and projected increases in precipitation (see Figure S5 for historical climate and Figure S6 and Figure S7 for future climate). Furthermore, several growth and development processes in bean as simulated by DSSAT have temperature optima in the range 20–35 °C, which means that they may be constrained by low temperatures under historical conditions in the Ethiopian highlands. While it is not possible to separate these processes and analyze them individually, as temperature rises these processes become less low-temperature constrained, leading to greater biomass accumulation and yield.

There are also areas within each of the three countries (for example, southern Tanzania, northern Uganda and eastern and western parts of Ethiopia) with crop yield reductions, especially where temperatures are high (minimum temperature near or above 20 °C) and the average precipitation is decreased up to 15%. Of special concern are the projected yield reductions in the north-western production hubs of Uganda, as well as specific pockets in the south-central production hubs in Tanzania.

### 3.2 Onset, duration, and intensity of stresses during the growing season for all countries and climate scenarios

**Fig. 4:**
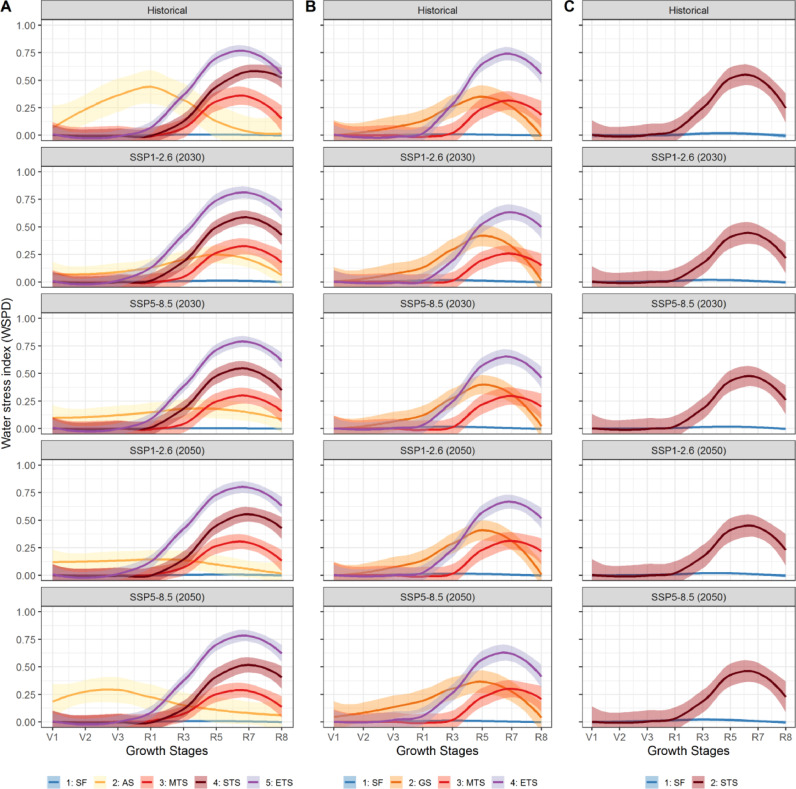
Water stress profiles in various physiological stages of plant growth for Tanzania (column A), Ethiopia (column B) and Uganda (column C). The first row is stress profiles under historical climate, and the remaining four rows are for future climate until 2030 under SSPs1–2.6, SSP2–4.5, SSP3–7.0 and SSP5–8.5, respectively. The x-axis of each plot shows growth stages and the y-axis shows probability. Only 25th and 75th percentiles of water stress are used and the loess method (Cleveland et al., 2017) is used to smoothen the lines. Each type of stress profile is shown by a unique color and name of each stress is written in legend on the bottom.

A total of six target population of environments (TPEs) were identified across the three countries, namely, stress-free conditions (SF), all-season stress (AS), moderate terminal stress (MTS), severe terminal stress (STS), extreme terminal stress (ETS), and grain filling stress (GS). Tanzania, Ethiopia, and Uganda can be divided into five, four and two TPEs, respectively (see Table 1).  Substantial variation is observed in the TPEs across countries and climate scenarios. Each of the TPEs is characterized by a unique seasonal pattern in the WSPD (Fig. 4, and Table S5). The pattern can be described according to its onset (when does it start?), duration (how long does it last?) and intensity (how dry does it get?).

**Table 1:** Total bean harvested area (‘000 ha) of all production hubs under various environmental groups based on the multi-model mean for each SSP. Country-specific percentage harvested area for each environmental group is shown in parentheses.

Country	TPEs^1^	Hist.	2030				2050			
			SSP1–2.6	SSP2–4.5	SSP3–7.0	SSP5–8.5	SSP1–2.6	SSP2–4.5	SSP3–7.0	SSP5–8.5
Uganda	SF	486.2 (84%)	384.2 (66%)	412.17 (71%)	404.95 (70%)	413.69 (71%)	380.1 (66%)	388.35 (67%)	368.03 (64%)	381.24 (66%)
	STS	92.6 (16%)	194.6 (34%)	166.62 (29%)	174.09 (30%)	165.1 (29%)	198.7 (34%)	190.45 (33%)	210.76 (36%)	197.56 (34%)
Ethiopia	SF	98.27 (61%)	102.55 (64%)	105.42 (65%)	108.48 (67%)	107.24 (66%)	108.93 (67%)	110.5 (68%)	101.88 (63%)	114.17 (71%)
	MTS	34.73 (21%)	23.11 (14%)	23.68(15%)	23.3(15%)	24.03 (15%)	20.2(12%)	18.73 (12%)	29.57 (18%)	22.39 (14%)
	ETS	8.09 (5%)	17.66 (11%)	13.62 (8%)	11.72 (7%)	14.09 (9%)	13.37 (8%)	16.41 (10%)	13.83 (9%)	11.78 (7%)
	GS	20.24 (13%)	18.01(11%)	18.61(12%)	17.83 (11%)	15.97(10%)	18.84 (13%)	15.69(10%)	16.06 (10%)	12.99(8%)
Tanzania	SF	462.2(59%)	436.84 (56%)	428.93 (55%)	449.57 (57%)	419.02 (53%)	402.34 (51%)	431.08(55%)	449.24(57%)	433.1 (55%)
	AS	145.9 (18%)	72.5 (9%)	57.04 (7%)	66.45 (9%)	73.69 (9%)	81.8 (11%)	61.15 (8%)	42.27 (5%)	42.83 (6%)
	MTS	108.54 (14%)	97.26 (12%)	103.11 (13%)	93.69 (12%)	107.79 (14%)	104.39 (13%)	105.13 (13%)	105.5 (13%)	95.74 (12%)
	STS	23.92 (3%)	92.66 (12%)	109.27 (14%)	88.09 (11%)	99.56 (13%)	110.1 (14%)	99.81 (13%)	90.55 (12%)	112.18 (14%)
	ETS	45.18 (6%)	86.5 (11%)	87.41 (11%)	87.95 (11%)	85.69 (11%)	87.13 (11%)	88.59 (11%)	98.2 (12%)	101.91 (13%)
Total	SF	1046.67	923.59	946.52	963	939.95	891.37	929.93	919.15	928.51
	AS	145.9	72.5	57.04	66.45	73.69	81.8	61.15	42.27	42.83
	GS	20.24	18.01	18.61	17.83	15.97	18.84	15.69	16.06	12.99
	MTS	143.27	120.37	126.79	116.99	131.82	124.59	123.86	135.07	118.13
	STS	116.52	287.26	275.89	262.18	264.66	308.8	290.26	301.31	309.74
	ETS	53.27	104.16	101.03	99.67	99.78	100.5	105	112.03	113.69

1 TPEs are as follows: SF= stress free; AS = all-season stress; MTS = moderate terminal stress; STS = severe terminal stress; ETS = extreme terminal stress; GS = grain filling stress; UGA= Uganda; ETH=Ethiopia; TZA=Tanzania.

Three of the TPEs (MTS, STS and ETS) show relatively similar seasonal behavior, peaking during the reproductive or grain filling period, and specifically after R5 (when 50% of plants begin developing seeds), but showing a gradient of severity, onset, and duration (Fig. 4, and Figure S8). ETS (extreme terminal stress) is the most intense and the longest, with an onset at the end of the vegetative period (V3, when plants have > 5 leaves), and a maximum intensity of 25% of crop potential transpiration being satisfied (i.e., WSPD=0.75) reached just before physiological maturity (R7). The second most severe stress is STS (severe terminal stress), which in the historical period onsets at R1 (anthesis) and peaks at R7 with around 30–40% of water demand satisfied. Lastly, the MTS (moderate terminal stress) onsets the latest (before R3, start of pod development), and peaks just before R7 with around 60% of water demand satisfaction.

The grain filling TPE (GS) shows a stress pattern starting very early (around V2, when plants have between 2 and 5 leaves), peaking at around WSPD=0.4 between beginning of flowering and maturity. Lastly, the all-season (AS) group, for which drought stress onsets with the start of the season, peaks at anthesis (R1, WSPD=0.46), and then decreases until water stress is negligible or zero.

### 3.3 Changes in water stress patterns across current and future climate scenarios

In the historical period, stress-free (SF) conditions show the greatest frequency across the three countries (59% in Tanzania, 61% in Ethiopia, and 84% in Uganda). In two of these (Uganda and Tanzania), however, the frequency of stress-free (SF) conditions decreases under future climate scenarios. Accordingly, the frequencies of the other TPEs, where various degrees of drought stress occur, increase in these two countries. In Uganda, the frequency of STS increases by more than two times under the future scenarios compared to the baseline. In Tanzania, terminal stresses (MTS, STS and ETS) are projected to become more frequent (50–70% increase), while the AS stress becomes less frequent (40–70% decrease) in future scenarios. The pattern of stress in the AS group changes substantially in the future scenarios, shifting its timing to be in the reproductive period in 2030, but also becoming much milder (the maximum value of WSPD during a growing season = 0.20 to 0.25).

By contrast, in Ethiopia, stress-free conditions are projected to increase from 61% in the historical climate to 64–67% by 2030 and to 63–71% by 2050. Terminal stresses (MTS and ETS) are and will be the dominant stresses in Ethiopia after SF conditions in terms of their frequency of occurrence, although at a reduced intensity compared to the historical period. Lastly, the grain filling TPE increases in intensity in the future especially in the early parts of the season.

### 3.4 Changes in water stress across production hubs

**Fig. 5:**
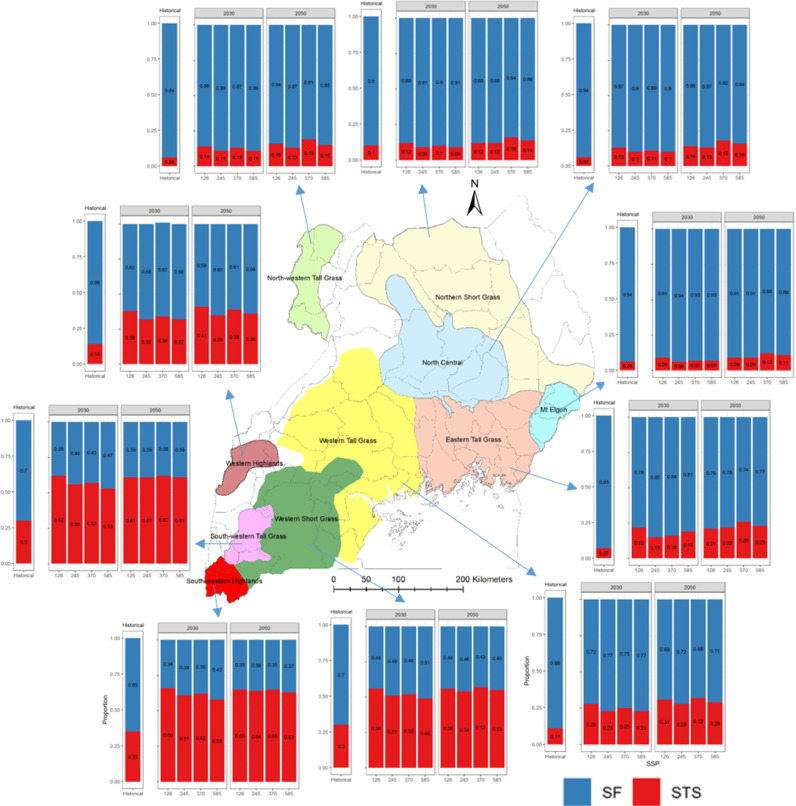
Production hubs in Uganda and water stresses in each bean production hub under present and future climate. The x-axis of each bar plot includes historical climate and future climate represented by four SSPs (SSP1–2.6, SSP2–4.5, SSP3–7.0 and SSP5–8.5) in two separate panels for 2030 and 2050. The y-axis shows proportion of each category of water stress. The water stress presented here is the average of the simulated water stresses obtained using inputs from five climate models for each SSP.

Drought stress is a major concern in the three-production hubs in Uganda (western short grass, south-western tall grass, and south-western highlands), which show up to a 33% probability of STS conditions in the historical period, with these probabilities doubling even considering the lowest emission scenario (i.e., SSP1–2.6, see Fig. 5, Table 2, Table S6).

**Table 2:** Bean production hub-specific changes in drought stress patterns in East Africa.

Country	Bean production hubs	Historical climate	Future climate
Uganda	Western short grass, south-western tall grass and south-western highlands	Around one third areas/years were under STS	STS is projected to double
	Remaining	< 15% of years/areas under STS	STS is projected to increase 2 to 4 times
Ethiopia	West Wellega and Pawe	Around 10–20% areas/years were under MTS and ETS	Negligible change in stress in West Wellega. A small decrease in stress in Pawe except in SSP3–7.0 in 2050.
	Western and Southern Rift Valley	Around one in every three areas/seasons are under stress. MTS is dominant in Western and GS in Southern Rift Valley	Up to 13% decrease in stress except in SSP3–7.0 in 2050.
	Central Rift Valley and Welkite and Silte	Following stresses have more than 50% probability of occurrence. 1. MTS, 2. ETS and 3. GS	Stresses decrease by 10–16%.
	Amhara and Haraghe	Stresses occur in around two thirds of areas/years. MTS is the dominant in both but the second most frequent is ETS in Amhara and GS in Haraghe.	Stresses decrease by 5–25%
	North East and North Shewa	Stresses occur around 80% of areas/years. MTS and ETS are the main stresses in North Shewa but in North East GS is also common.	Stresses decrease by 10–20%
Tanzania	Kagera, Kigoma and Mpanda	Most often (75% time) drought stress-free. GS is the main stress.	Most often (75% time) drought stress-free. MTS is the main stress.
	Southern Highlands, Southern Lowlands and Tanga	More than half of the time drought stress-free.	More than half of the time drought stress-free. STS and ETS will become more important.
	Shinyanga, Maara, Northern Highlands, Northern Semi-arid Highlands and Southern Mid-altitude	More than half of the time under drought stress.	Frequency of drought stresses increases. GS becomes less important in Shinyanga and Maara. ETS becomes more important in the highlands.

^1^SF= stress free; AS = all-season stress; MTS = moderate terminal stress; STS = severe terminal stress; ETS = extreme terminal stress; GS = grain filling stress.

In the remaining production hubs, the probability of drought stresses is less frequent both under present and future climate. These results suggest that a differentiated breeding strategy is warranted for southern Uganda whereby selection under terminal stress conditions should be performed. Selection under drought stress-free conditions would be the priority for the rest of the production hubs.

**Fig. 6:**
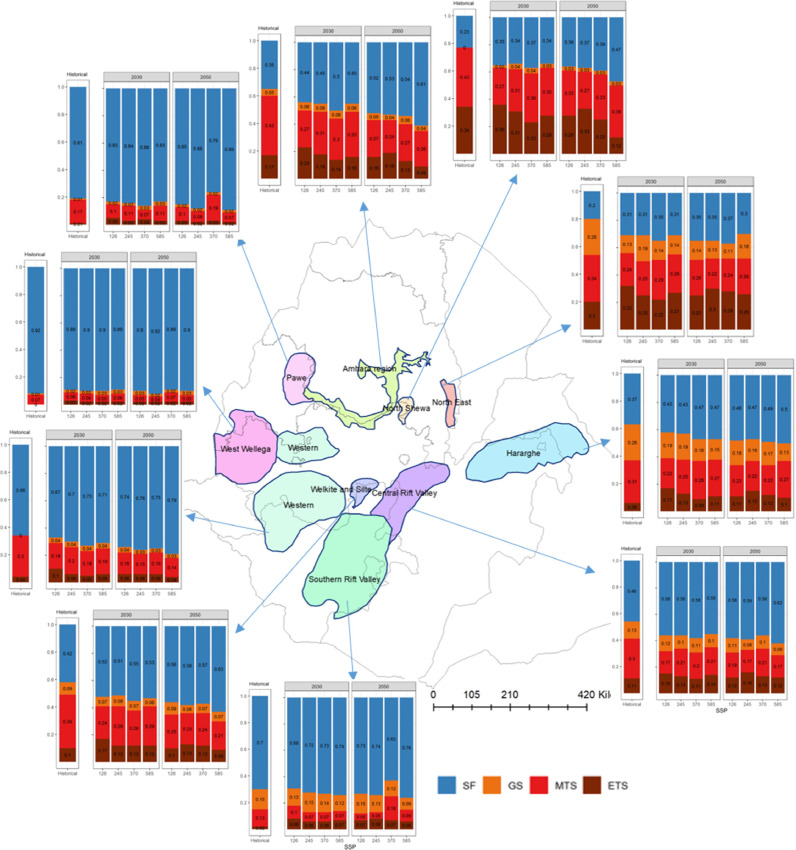
Same as Fig. 5 but for Ethiopia.

In Ethiopia, six out of 10 production hubs appear with particularly high frequencies of water stress conditions, namely, the “Amhara Region”, “North Shewa”, “North East”, “Haraghe”, “Central Rift Valley”, and “Welkite and Silte” (Fig. 6, Table 2, Table S6). For these areas, the bean program should focus its efforts on breeding for terminal drought stress, aiming at selecting genotypes that can withstand both moderate and extreme drought (whereby water supply is 30–70% lower than required by the crop) onsetting right after anthesis and staying relatively high until physiological maturity. Together, these two TPEs account for 37–77% of the conditions in these production hubs. The remaining production hubs either show relatively low probability of drought stress, especially under climate change (e.g. those in the western parts) or appear to have a variety of stresses occurring during the season at different likelihood levels. It is noteworthy that while decreasing in importance, an explicit focus on drought should be kept as a target for breeding.

**Fig. 7:**
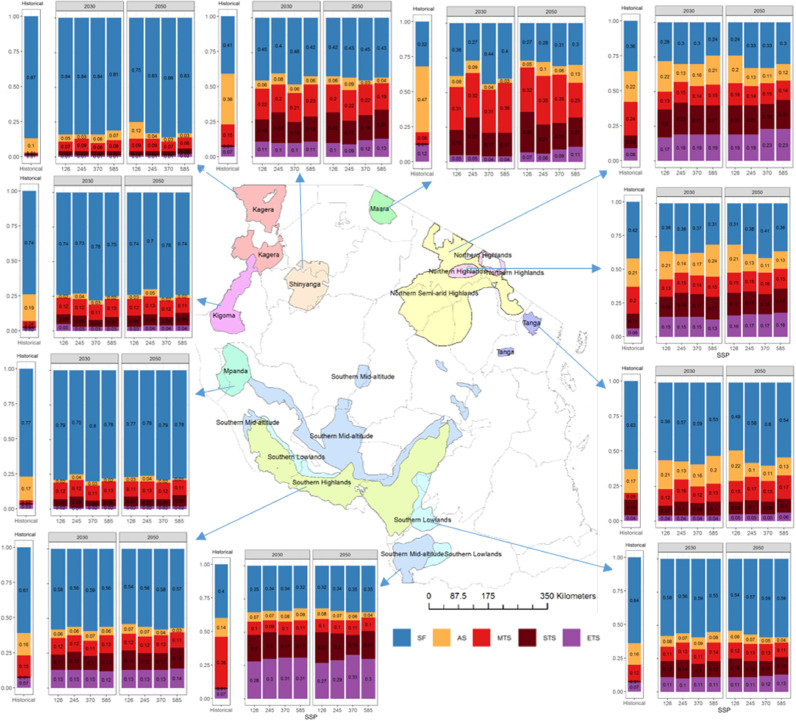
Same as Fig. 5 but for Tanzania. Five categories of stresses exist in Tanzania — SF (stress-free) in blue color, AS (all season) in yellow color, MTS (moderate terminal stress) in red color, STS (severe terminal severe) in maroon color and ETS (extreme terminal stress) in purple color.

In Tanzania, the five production hubs (Shinyanga, Maara, Northern Highlands, Northern Semi-arid Highlands and Southern Mid-altitude) show relatively high frequencies of water stressed TPEs in historical climate and the stress is projected to increase further in future climates (Fig. 7, Table 2, Table S6). Across these production hubs, these TPEs vary in both space and time. In Shinyanga and Maara, historical conditions indicate that the primary TPE is the all-season (AS) stress, whereas MTS dominates in future. In highland environments, ETS, and to a lesser extent STS, are projected to dominate under future climate scenarios. Extreme terminal drought stress is also projected to dominate in the Southern Mid altitude, with around two third of the production hub under water stress in future. The remaining production hubs were stress-free 40–75% time in the historical climate and are projected to remain so in the future climate. The complexity and variation in TPEs across Tanzanian production hubs suggest that a suitable target for the breeding program would be broad adaptation to drought.

### 3.5 Variation in minimum temperatures across environment groups and bean production hubs

**Fig. 8:**
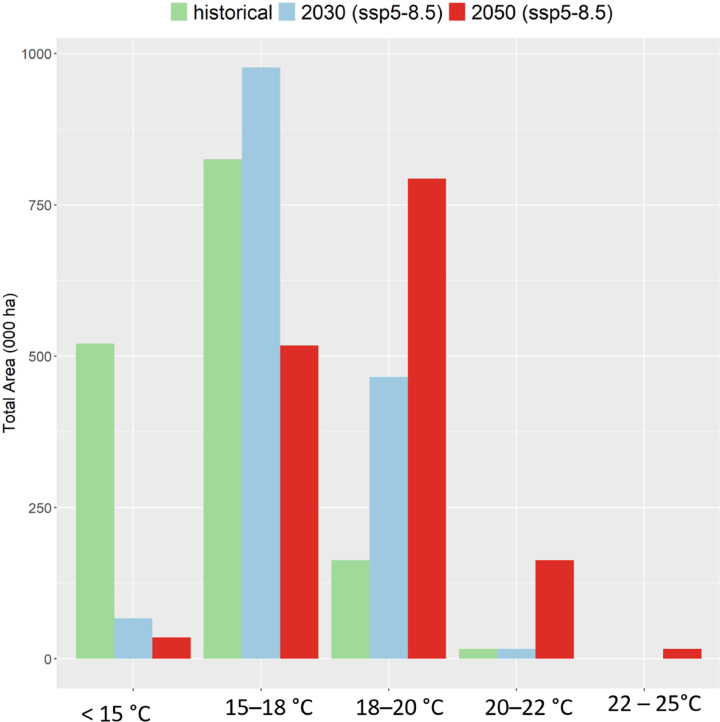
Thermal environments based minimum temperature (T_min_) during historical (1991–2010), near future (2030) and far future (2050) scenarios. The thermal environments were determined using fixed classes defined jointly with the crop improvement team.

Based on the average minimum temperature (*T_min_*) experienced by the bean crop during growing seasons, the productions hubs in Ethiopia are divided into three thermal environments (TEs), Tanzania into four TEs and Uganda contains the five TEs (Figure S9, S10). Overall, the vast majority of bean growing areas experience *T_min_* < 18 °C in the historical period (Fig. 8). Climate change shifts the temperature distribution so that by 2030 and 2050 most of the bean area experiences *T_min_* > 18 °C. Despite these changes, only a small amount of area experiences temperatures greater than 22 °C by 2050. Since there is virtually no occurrence of heat stress, this implies no co-occurrence of heat stress jointly with drought (see Table S7). The geographic distribution of future thermal environments suggests that only in Uganda heat stress is a concern under future scenarios.

## 4 Discussion

### 4.1 Future changes in drought stress and yield and their implications for bean breeding

Drought stress experienced by a bean crop during a growing season results from the interactions between precipitation, temperature, properties of soil, the atmospheric CO_2_ concentration, and the genetic properties of a cultivar*.* Therefore, drought tolerant cultivars cannot be realized without understanding these interactions. Since, it takes a decade or more from the design to adoption of a cultivar for commercial production (see e.g., Challinor et al., 2016), it is necessary to understand the types of drought stress and their seasonal variation for a given environmental group not only in the present climate but also in the future climate. Here, we have grouped this variation in the historical, near-future (2030) and far-future (2050) climate, for Ethiopia, Tanzania, and Uganda into six different environmental groups. The results suggest that selection for drought stress tolerant genotypes should be a priority for the three countries (see Table 2 for specific priorities).

It is relatively well documented that drought is and will continue to be a critical constraint for bean production (see Beebe et al., 2013a; Hummel et al., 2018; Katungi et al., 2009), and our study contributes to determining environmental groups for drought and their frequencies across bean production areas. The finding from this study that drought stress is projected to become more frequent (in Uganda and Tanzania) but less intense (in Ethiopia and Uganda) in future under climate change is in agreement with Heinemann et al. (2017) for the rainfed common bean production systems in Brazil. Based on this result, these authors suggest that the breeding program should perform weighted selection using drought stress profile frequencies as weights. For Ethiopia and Uganda, this is a likely suitable breeding strategy for national programs. For Tanzania, the complexity of the TPEs and their geographical distribution suggests that broad adaptation to drought is potentially a more viable strategy. Furthermore, our results show that minimum temperature increases will likely pose important constraints to bean production only in drought stress free environments in Uganda, and to a lesser extent in southern Tanzania. We underscore importance of developing a climate-tailored strategy, since the regions that are at the margins of suitable areas could become less suitable as temperature rises (Beebe et al., 2011; Ramirez-Cabral et al., 2016) (also see yield declines in Fig. 3).

**Fig. 9:**
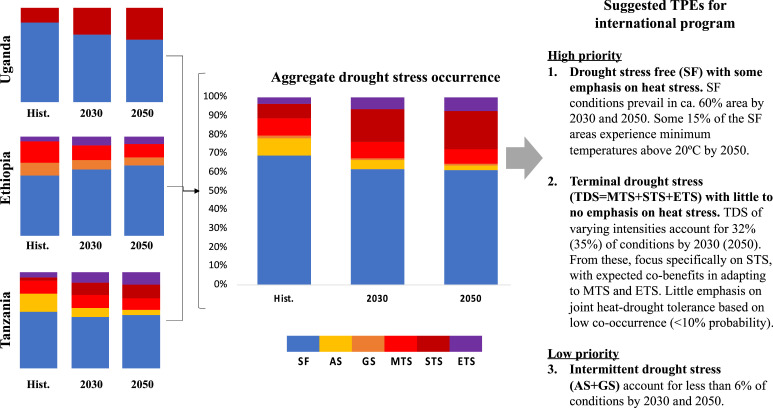
Aggregation of country level priorities to derive regional TPEs for the international bean crop improvement program in the CGIAR.

The findings from this study can add value to the existing drought tolerance breeding programs. Bean breeding is currently done at the international level by the CGIAR (Beebe, 2012; Beebe et al., 2011), and subsequently at the national level by breeding programs within the National Agricultural Research Systems (NARS). Hence, priority target population of environments (TPEs) are needed at both levels. At the country-level, the six TPEs found here (see Fig. 5, Fig. 6, Fig. 7) and the changes in temperature environments are useful for national bean crop improvement programs to prioritize their actions and tailor their strategies to the range of stresses projected to occur by 2030 and 2050 in their respective countries. For the international breeding program, based on the relative importance of specific stresses across the three countries, their similarities, the knowledge of the bean breeders, and the changes in frequency projected under future scenarios, we propose that the international breeding program focuses on two major TPEs in East Africa (see Fig. 9). Firstly, a drought stress-free TPE with some emphasis on heat stress; and secondly, a terminal drought stress (TDS, aggregating MTS, STS, and ETS) TPE with little to no emphasis on heat stress. A last TPE of low priority, AS and GS combined, (<6% probability historically and in the future) is identified in which drought stress is prevalent throughout the season. Nevertheless, the definition of TPE is a dynamic process that will be modified as additional parameters are included in the analysis.

Apart from setting priorities at international and national levels, these results can also be helpful to improve drought protocols in the breeding trials and locate multi-location trials, needed for testing the genotypes of mid and advanced generation materials. The breeding program can mimic the specific details of drought stresses projected for 2030 and 2050 including their onset, intensity, and duration during a growing season, for example through rain shelters, and test genotypes under drought and high temperature conditions in greenhouse experiments or in hot environments (e.g., CIAT, 2015; Suárez-Salazar et al., 2018).

### 4.2 Limitations and future work

This study used a well-established crop model, calibrated and evaluated for a representative cultivar for East Africa, and used an ensemble of climate model projections from the latest Intergovernmental Panel on Climate Change (IPCC) climate model ensemble (i.e., CMIP6). We use field data for model calibration and evaluation, as well as to define crop management options including planting dates in consultation with a team of experts. Despite these strengths, several avenues for future work exist that can improve on or extend our results. Firstly, the results from this study are based on only one genotype, cv. Calima, but farmers in Ethiopia, Tanzania and Uganda use various other cultivars, and there is turnover in these cultivars over time. Drought stress pattern and their yield impacts may differ for different cultivars, given differences in their nature (bush or climber types), root characteristics, water use efficiency, cycle duration, and other drought tolerance traits. In Ethiopia, a significant proportion of production is with cultivars of the Mesoamerican gene pool, which often present greater degrees of drought tolerance. Future work should seek to update this study using a larger number of varieties with different drought tolerance levels and crop cycle lengths, exploring variations in the resulting TPEs, but also investigating genotype targeting within and across the TPEs and bean production hubs.

Other aspects that merit future work include the consideration of other stresses that are of importance in bean growing areas. Low soil fertility including deficiency of Nitrogen (N), Phosphorus (P) and Potassium (K) along with toxicity from Aluminum and Manganese are prevalent in bean growing areas in Africa (Thung and Rao, 1999). Likewise, soil compaction is a major constraint for bean production (Thung and Rao, 1999), as it can limit bean growth, development, and yield (Buttery et al., 1998) by reducing infiltration, air flow and extraction of water and nutrients (Descalzi et al., 2018; Kozlowski, 1999). Waterlogging, which is expected to increase in East Africa, can cause anoxia to bean roots, and can limit both symbiotic N_2_ fixation and N uptake resulting into reduced root growth and nodulation (Beebe et al., 2013b). Pests and diseases also limit bean production in East Africa and elsewhere, and existing studies suggest that climate change could increase the severity and spread of pathogens, and insect pests (Bebber et al., 2014; Beebe et al., 2013b; Chaloner et al., 2021). Future work could seek to consider all these stresses and their interactions with drought stress, yet the complexity of these stresses and their interactions requires substantial progress in bean crop physiology and model development.

## 5 Conclusions

The frequency, intensity, duration, and timing of drought stress in Uganda, Ethiopia, and Tanzania will change significantly as a result of climate change. Based on the results presented here, we conclude common bean crop improvement should maintain an explicit focus on drought. At the national level, Ethiopia and Uganda could implement weighted selection schemes in which the frequencies of drought environments are used as weights. For Tanzania, broad adaptation to drought is warranted. Only in Uganda, and in a small part of southern Tanzania, we document some levels of heat stress. At the international level, we identify two major TPEs, namely, a drought stress-free TPE with some emphasis on heat stress; and a terminal drought TPE with little to no emphasis on heat stress.

## Author statement

**PJ**: Data analysis, Visualization, First draft preparation; **PJ and JRV**: Conceptualization, Methodology, Investigation, manuscript reviewing and editing**; JRV:** Supervision; **SB, CM**: manuscript reviewing and editing; **PAT**: Model calibration and evaluation.

## Declaration of Competing Interest

The authors declare the following financial interests/personal relationships which may be considered as potential competing interests: Prakash Kumar Jha reports financial support was provided by Accelerated Varietal Improvement and Seed Delivery of Legumes and Cereals in Africa (AVISA). Prakash Kumar Jha reports a relationship with International Center for Tropical Agriculture that includes: employment. Steve Beebe reports a relationship with International center for Tropical Agriculture (CIAT) that includes: employment. Patricia Alvarez-Toro reports a relationship with International center for Tropical Agriculture (CIAT) that includes: employment. Clare Mukankusi reports a relationship with International center for Tropical Agriculture (CIAT) that includes: employment. Julian Ramirez-Villegas reports a relationship with International center for Tropical Agriculture (CIAT) that includes: employment. None

## Data Availability

Data will be made available on request.

## Appendix Supplementary materials

Image, application 1
