# Supplementary material for: Characterizing patterns of seasonal drought stress for use in common bean breeding in East Africa under present and future climates
Source: Agric For Meteorol. 2023 Nov 15;342:109735. doi: 10.1016/j.agrformet.2023.109735 (PMC10636599; doi:10.1016/j.agrformet.2023.109735)
Supplement: Supplementary file 1 [file mmc1.docx]

**Supplementary Information**

**Characterizing patterns of seasonal drought stress for use in common bean breeding in East Africa under present and future climates**

**Authors**

Prakash K. Jha^1,2^^[[1]](#footnote-1)^ , Steve Beebe^1^, Patricia Alvarez-Toro^1^, Clare Mukankusi^6^, Julian Ramirez-Villegas ^1, 3, 4,5^

**Affiliations**

^1^ International Center for Tropical Agriculture (CIAT), Km 17, Recta Cali-Palmira, Cali, Colombia

^2^ Division of Agriculture and Natural Resources, University of California, Merced, CA, United States

^3^ Bioversity International, Via di San Domenico, 1, 00153, Rome, Italy

^4^ CGIAR Research Program on Climate Change, Agriculture and Food Security (CCAFS), c/o CIAT, Palmira, Colombia

^5^ Plant Production Systems Group, Wageningen University and Research, Wageningen, The Netherlands

^6^ International Center for Tropical Agriculture (CIAT), P. O. Box 6247, Kampala, Uganda

**Supplementary Text S1: Model calibration and evaluation**

The field trials for model calibration and evaluation were conducted at Villanueva Municipality in the department of Santander (Colombia), (latitude 6°36'38'' N and longitude 73° 8' 58'' W), located at an altitude of 1,554 meters above sea level. To understand how representative is Villanueva with respect to East African bean growing environments, we performed a climatic similarity analysis. Climatic similarity was computed following Ramírez-Villegas et al. (2011) and Aggarwal et al. (2018) who use a normalized Euclidean distance to assess climatic similarity from monthly precipitation and temperature data^[[2]](#footnote-2)^. We use the default parameters, whereby temperature and rainfall are given equal weights and calendar rotation is done based on rainfall. Fig. S1 shows the climatic similarity of Villanueva (Santander) compared to the three East African countries we study (Ethiopia, Uganda, Tanzania). We find that while the climatic similarity varies across the geographic space, bean growing environments (i.e., bean corridors depicted as blue polygons in Fig. S1) show high levels of climatic similarity compared to areas that do not grow beans. Furthermore, the majority of areas that grow beans have a similarity score of 0.7 (considered as high similarity). Therefore, we conclude that model calibration and evaluation for cv. Calima using data from Villanueva will produce results that also apply over East African bean growing environments.

Figure S1. Climatic similarity of Villanueva (Colombia) across East Africa. Dashed blue polygons are bean corridors as reported by Farrow and Muthoni-Andriatsitohaina (2020). The climatic similarity varies from 0 (highly dissimilar) to 1 (highly similar).

The calibration process included two steps: (1) sensitivity analysis, and (2) parameter calibration. Sensitivity analysis was done given the multi-dimensional nature of the calibration problem (i.e., too many parameters, sometimes affecting each other), and the fact that each parameter typically has a different effect on relevant crop model outputs (e.g., LAI, yield). Sensitivity analysis (SA) thus allowed us to identify the most relevant parameters for calibration. The most relevant parameters were then calibrated using a genetic algorithm approach.

The sensitivity analysis was performed by first defining minimum and maximum values of all cultivar parameters (15 parameters in CROPGRO-DRYBEAN), and then drawing 10,000 parameter samples following a Latin Hypercube Sampling (LHS) design, with uniform probability distributions for all parameters. The model was then run for each of the 10,000 samples for 3 sites, representing a range of bean growing environments. The simulated output (i.e., days to flowering, days to maturity, leaf area index, yield and total biomass) was then used to partition the variance stemming from each parameter using the Sobol 2007 methodology (Sobol’ et al., 2007). Variance partitioning helped identify the fractional contribution of each parameter to the total variance in simulated output. Only parameters with a total contribution of more than 5% in at least one site were considered for calibration.

Calibration was then performed via a genetic algorithm. Genetic algorithms have been extensively used in modelling to calibrate model parameters, especially where the dimensionality of the parameter space is significant, and computational efficiency is required (Guo et al., 2021; Jones et al., 2011; Li et al., 2018). Here, we sequentially drew sequential parameter samples at random following a uniform probability distribution, ran the model, and then retained or discarded the new parameter set. More specifically, at each sample, the model was run, and the simulated output (yield, total biomass, LAI, days to flowering, days to maturity) compared to observations using the root mean square error (RMSE). The resulting RMSE was compared with the RMSE of the immediately prior sample, and the new sample retained (discarded) if the RMSE was lower (greater). The process was repeated until the RMSE was sufficiently low (10% of the observations, normalized by the mean), or until 10,000 samples were drawn (RMSE gains after 10,000 samples offered negligible gains). The final cultivar parameters from this exercise are shown in Table S1. The model was able to perfectly reproduce the anthesis day for the 1^st^ planting date but the error ranged from 3 to 14 days in the case of other phenology variables (Table S2).

Table S1. The final model parameters of the cv. Calima obtained from the calibration process. CROPGRO-DRYBEAN cultivar parameters not listed here were left as default.

| **Parameter** | **Value** |
| --- | --- |
| Ecotype (ECO#) | ANDDET |
| Critical short day length below which reproductive development progresses with no daylength effect (for shortday plants) (hour) (CSDL) | 12.17 |
| Slope of the relative response of development to photoperiod with time (positive for shortday plants) (1/hour) (PPSEN) | 0.0 |
| Time between plant emergence and flower appearance (R1) (photothermal days) (EM-FL) | 26.7 |
| Time between first flower and first pod (R3) (photothermal days) (FL-SH) | 3.0 |
| Time between first flower and first seed (R5) (photothermal days) (FL-SD) | 14.9 |
| Time between first seed (R5) and physiological maturity (R7) (photothermal days) (SD-PM) | 16.11 |
| Time between first flower (R1) and end of leaf expansion (photothermal days) (FL-LF) | 10.00 |
| Maximum leaf photosynthesis rate at 30 C, 350 vpm CO2, and high light (mg CO_2_/m^2^-s) (LFMAX) | 1.07 |
| Specific leaf area of cultivar under standard growth conditions (cm^2^/g) (SLAVR) | 276.15 |
| Maximum size of full leaf (three leaflets) (cm^2^) (SIZLF) | 151.3 |
| Maximum fraction of daily growth that is partitioned to seed + shell (XFRT) | 1.00 |
| Maximum weight per seed (g) (WTPSD) | 0.96 |
| Seed filling duration for pod cohort at standard growth conditions (photothermal days) (SFDUR) | 15.0 |
| Average seed per pod under standard growing conditions (#/pod) (SDPDV) | 3.50 |
| Time required for cultivar to reach final pod load under optimal conditions (photothermal days) (PODUR) | 10.0 |

Table S2. The measured and simulated values of the variables for cv. Calima from the model **calibration exercise**.

| Variables | Planting Date 1 (P1) | | | Planting Date 2 (P2) | | |
| --- | --- | --- | --- | --- | --- | --- |
|  | Simulated | Measured | Difference (Sim-Meas) | Simulated | Measured | Difference (Sim-Meas) |
| Anthesis day (dap) | 38 | 38 | 0 | 39 | 34 | 5 |
| First pod day (dap) | 42 | 45 | -3 | 43 | 39 | 4 |
| First seed day (dap) | 57 | 62 | -5 | 61 | 52 | 9 |
| Physiological maturity day (dap) | 74 | 70 | 4 | 79 | 65 | 14 |

Apart from these variables, we also analyzed the model’s capacity to capture the time-progress of Leaf Area Index (LAI), weight of dry leaves (LWAD), above-ground dry weight of plants (CWAD), stem dry weight (SWAD) and dry weight of grain (GWAD) over the growing season. The model performs well at capturing dynamic progress in LAID, SWAD and LWAD, although progress of LAI in the later part of growing season (Fig S2 a, e) shows some discrepancies between model and observations. Likewise, we observe differences between simulations and observations for total aboveground biomass (CWAD) after development of pod (around 60 days after planting). The model underestimated yield, with measured yields for 1^st^ and 2^nd^ treatments being 2,294 kg ha^-1^ and 2,442 kg ha^-1^, while the simulated yields were 1,659 kg ha^-1^ and 1,406 kg ha^-1^ respectively.


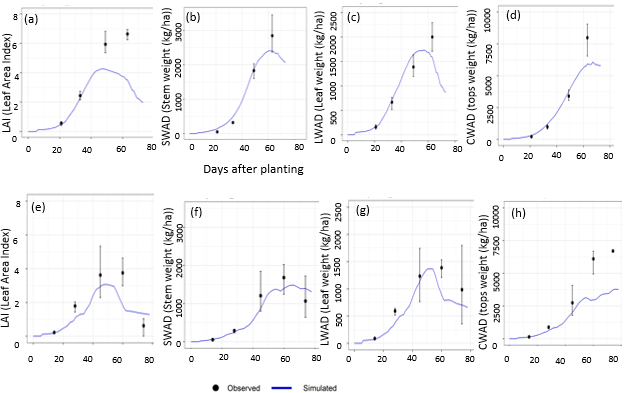


Figure S2. Observed and model simulated values of the leaf area index (a), stem weight (b), leaf weight (c), tops weight (d) over the growing season with the final modal parameters from the calibration exercise for the trial sown on first planting date (P1). Same but for the trial on second planting date (P2) are in (e), (f), (g) and (h). The x-axis shows the days after planting and y-axis represents the units of the respective variables. The blue color line is the model data and the black dots is for the measured data. The error bar represents the range of the data from replications.

We evaluated the model performance using trial data from the same site but for two different sowing dates in 2014 (Figure S3, and Table S3).
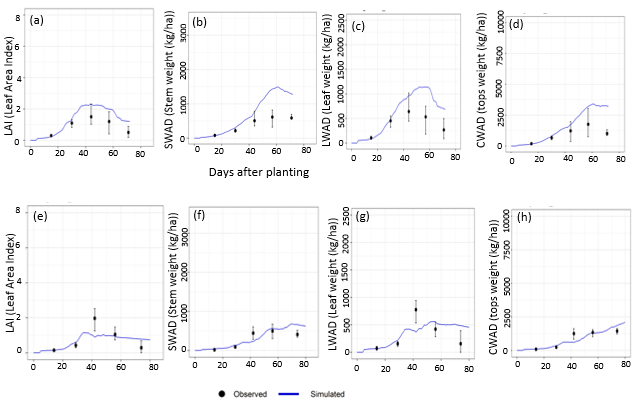


Figure S3. Same as Fig S2 but for the validation exercise. The top panel includes figures for the trial of 3^rd^ planting date (P3) and bottom panel shows figures for the trial of fifth planting date (P5) (bottom row).

Table S3. The measured and simulated values of the variables for cv. Calima from the **validation exercise from same site but for different planting dates**.

| Variables | Planting Date 3 (P3) | | | | Planting Date 5 (P5) | | | |
| --- | --- | --- | --- | --- | --- | --- | --- | --- |
|  | Simulated | Measured | Difference (Sim-Meas) | Simulated | | Measured | Difference (Sim-Meas) |  |
| Anthesis day (dap) | 38 | 33 | 5 | 40 | | 33 | 7 |  |
| First pod day (dap) | 42 | 37 | 5 | 44 | | 37 | 7 |  |
| First seed day (dap) | 58 | 54 | 4 | 62 | | 48 | 14 |  |
| Physiological maturity day (dap) | 73 | 72 | 1 | 81 | | 64 | 17 |  |
| Yield (kg ha ^-1^) | 654 | 525 | 129 | 863 | | 618 | 245 |  |

As an additional benchmark to compare our results, Table S4 shows phenology model evaluation results from Alvarez-Toro et al. (2020) for the same cultivar (cv. Calima) but at the Altobonito farm, Vereda los Tendidos, Municipality of Popayán. These results are consistent with ours in terms of model performance and model error.

Table S4. The measured and simulated values of the variables for cv. Calima **from the study of Alvarez-Toro et al. (2020)**.

| Variables | Simulated | Measured | Difference (Sim-Meas) |
| --- | --- | --- | --- |
| Anthesis day (dap) | 37 | 39 | -2 |
| First pod day (dap) | 42 | 45 | -3 |
| First seed day (dap) | 50 | 62 | -12 |
| Physiological maturity day (dap) | 77 | 80 | -3 |


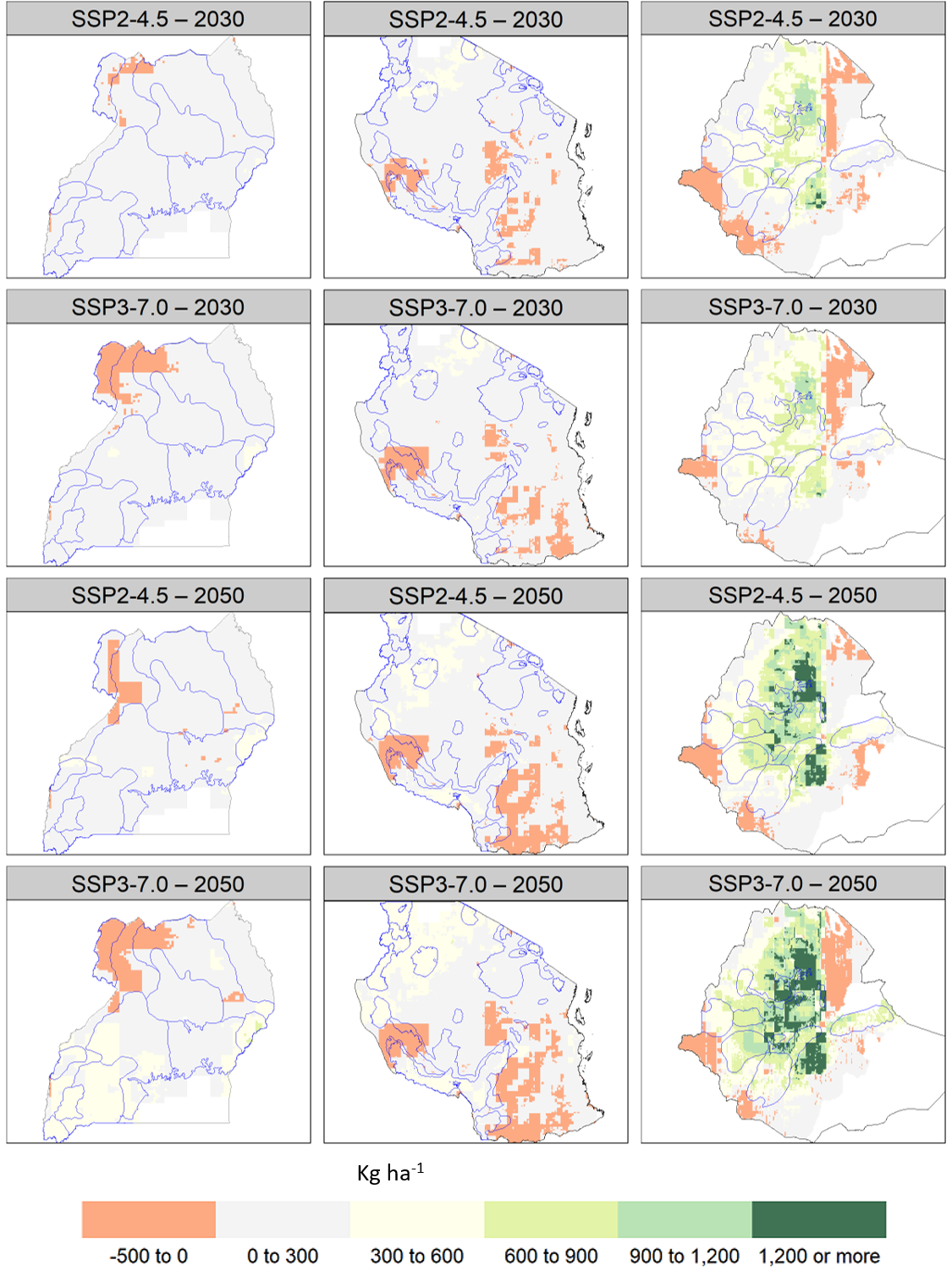


Figure S4. Same as Figure 3 in the main text but for SSP2-4.5 and SSP3-7.0.


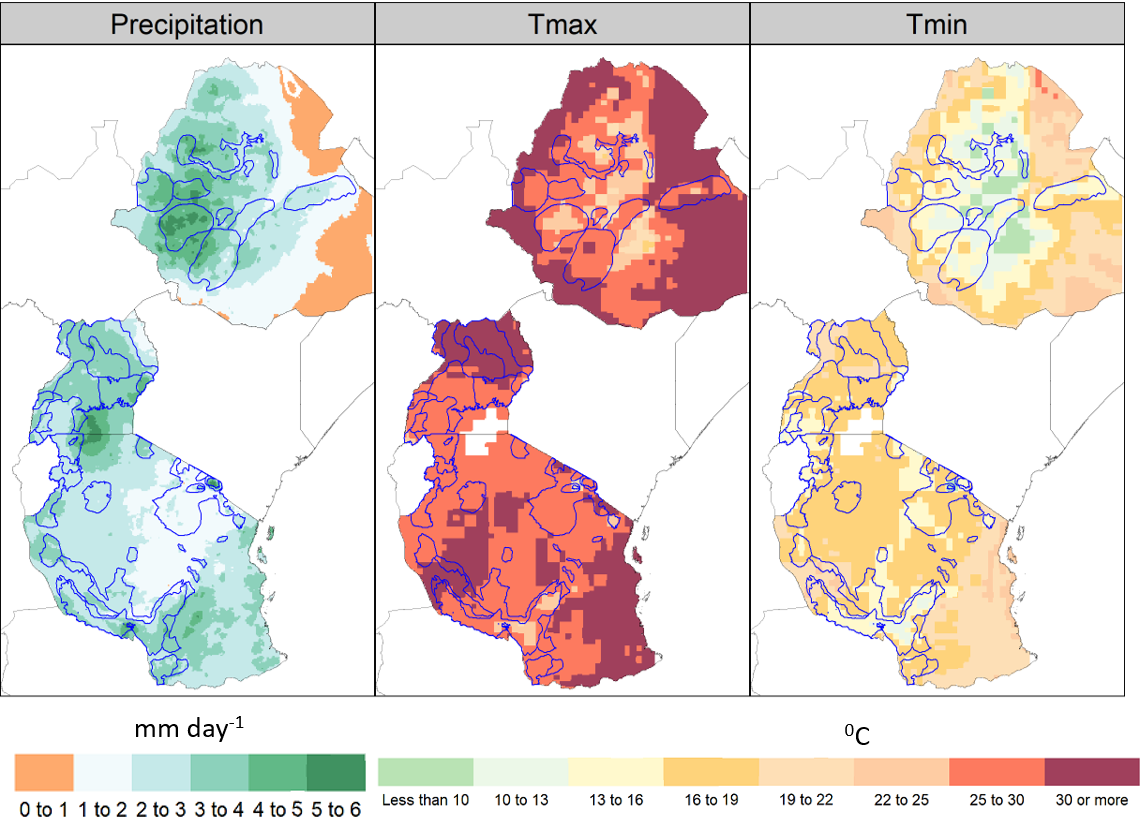


Figure S5. Average precipitation (left), daily maximum temperature (middle) and daily minimum temperature (right) in Ethiopia, Uganda and Tanzania, during the historical period 1991–2010. The thin grey polygons represent different bean corridors.


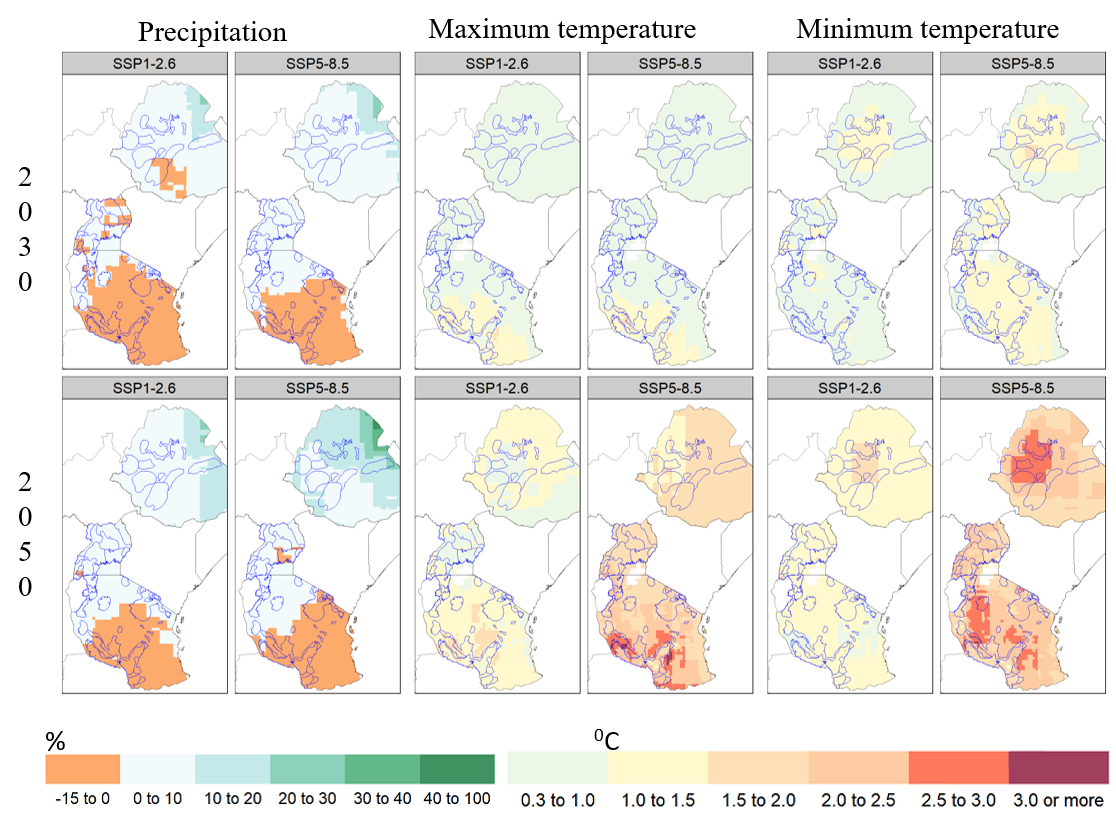


Figure S6. Projected changes in average precipitation (1^st^ and 2^nd^ columns from left), daily maximum temperature (3^rd^ and 4^th^ columns) and daily minimum temperature (5^th^ and 6^th^ columns) in Ethiopia, Uganda and Tanzania, for the period 2021–2040 (the top ows) and 2041–2060 (the bottom 2 rows), relative to 1991–2010. The thin blue polygons represent different bean production hubs. Baseline climate conditions are shown in Supplementary Fig. S3.


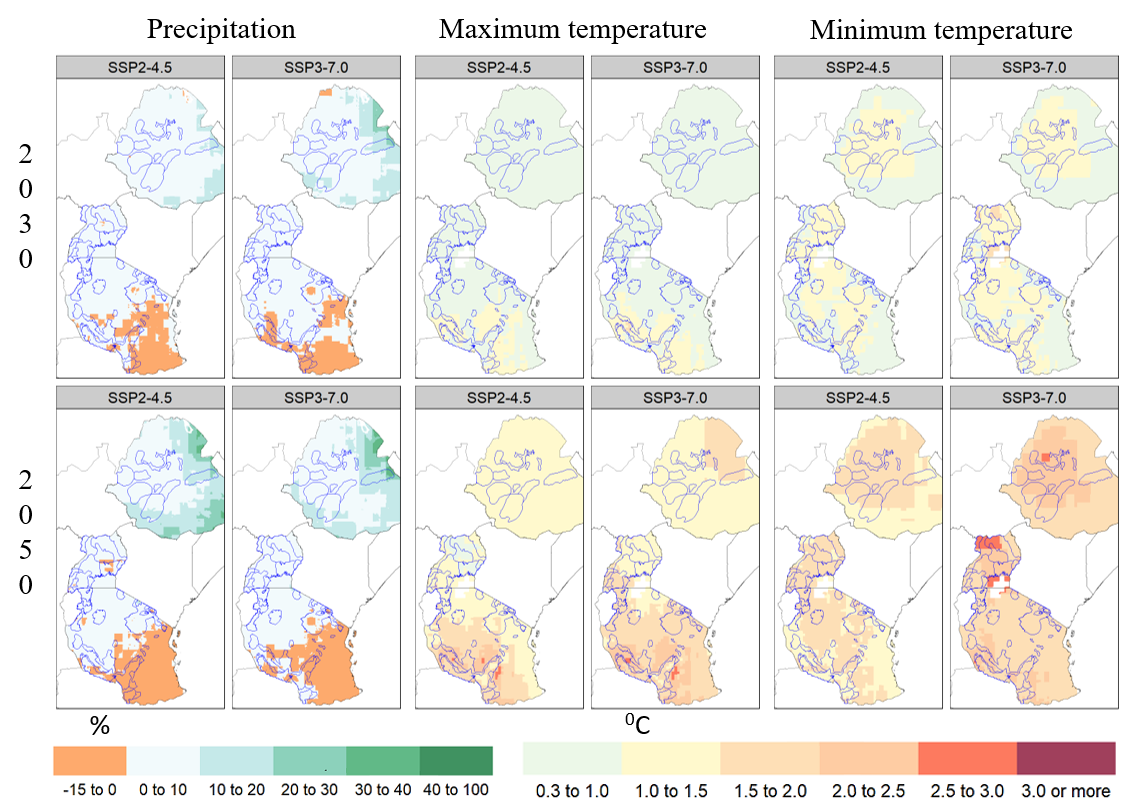


Figure S7. Same as Figure S6 except for SSP2-4.5 and SSP3-7.0.


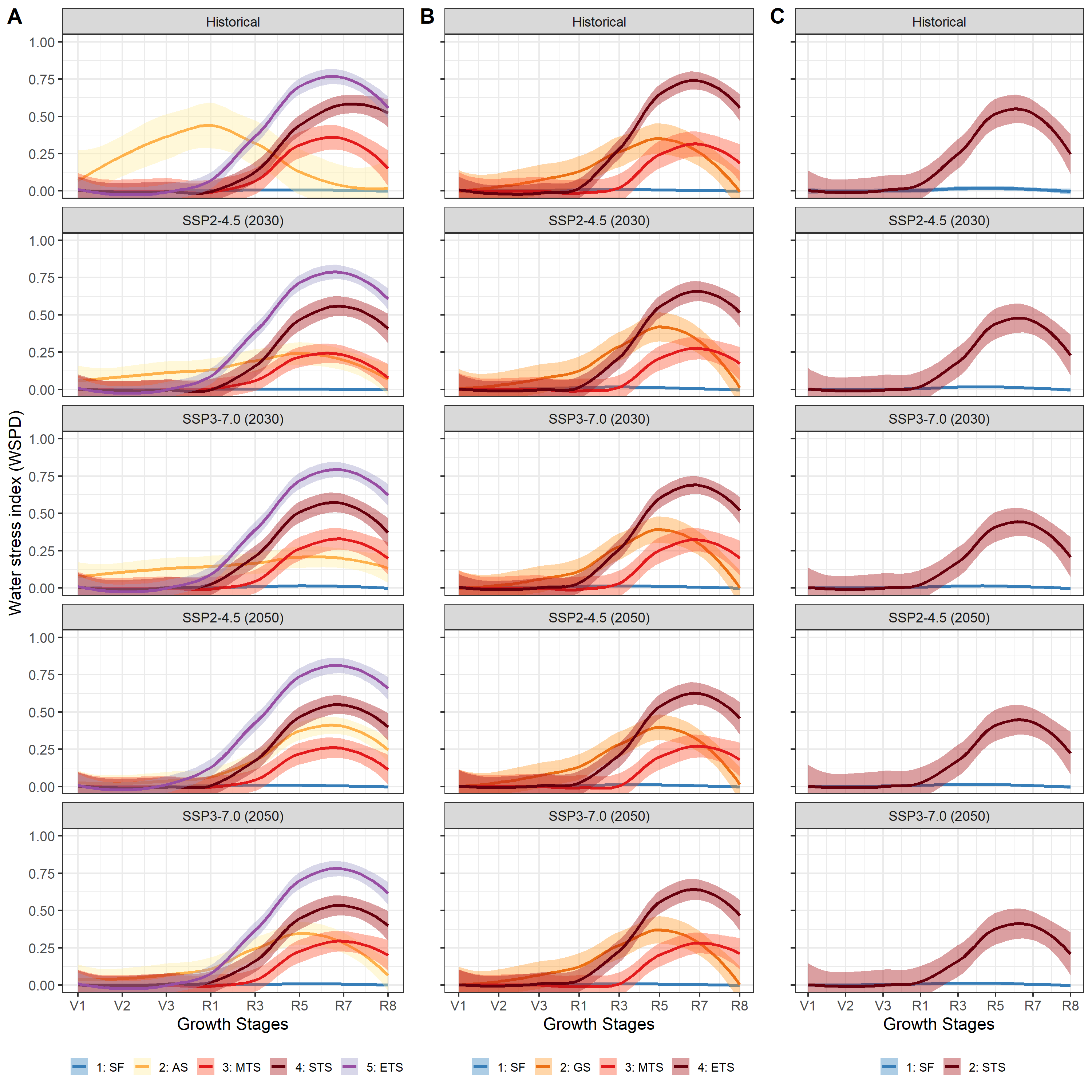


Figure S8. Same as Figure 4 in the main text but for SSP2-4.5 and SSP3-7.0.


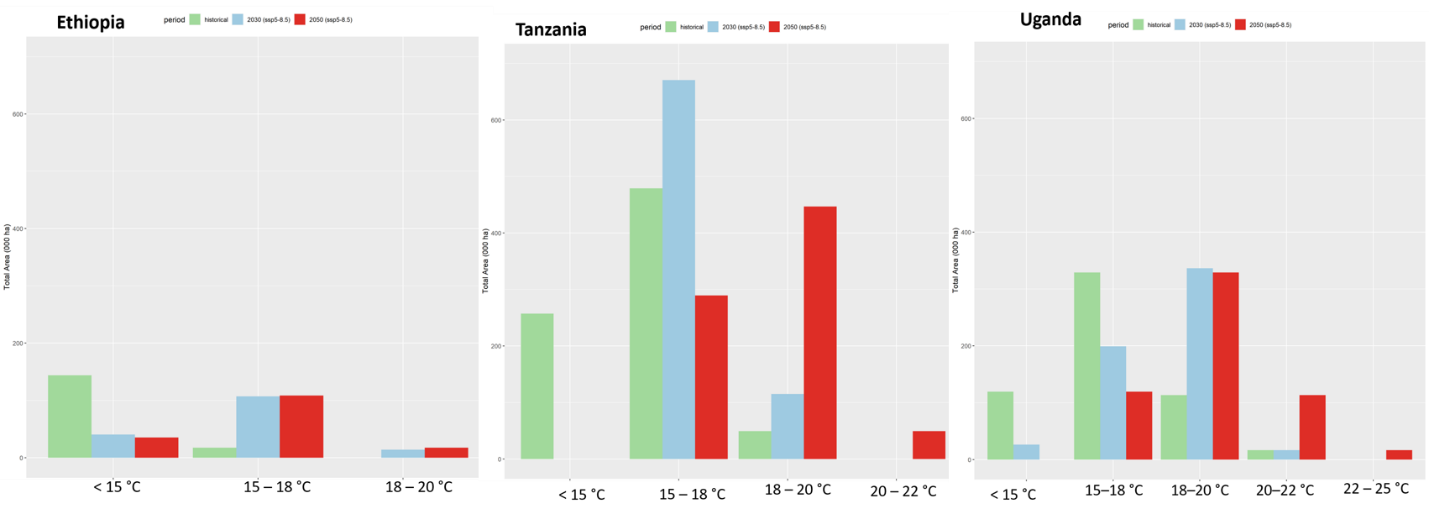


Figure S9. Same as Figure 8 in the main text but for individual countries — Ethiopia (in the left), Tanzania (in the middle) and Uganda (in the right).


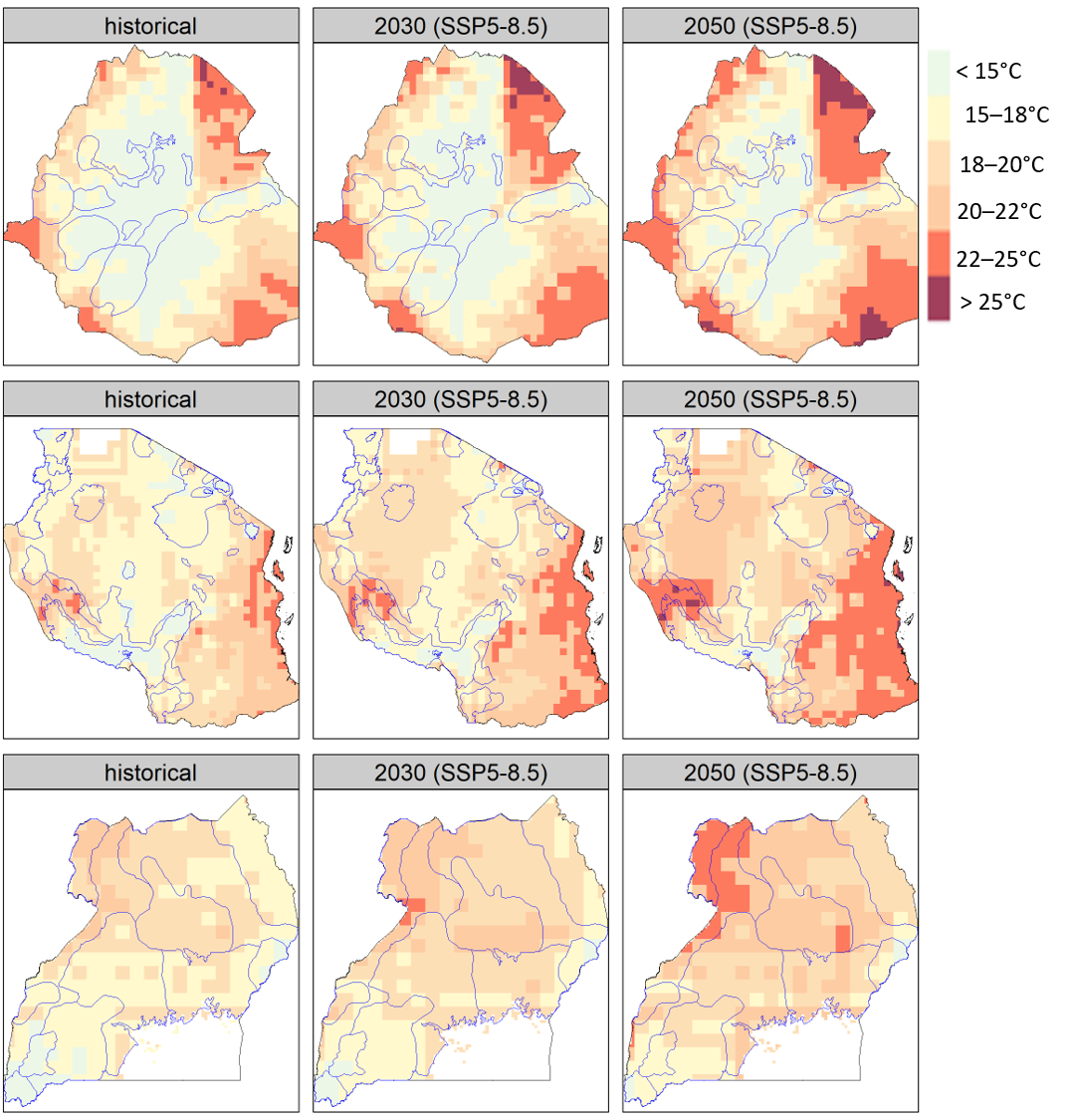


Figure S10. Map of thermal environments based minimum temperature (T_min_) during historical (1991–2010) period in the first column, near future (2030) in the middle column and far future (2050) in the right column. Ethiopia, Tanzania, and Uganda are in the first, second and third rows respectively. The thermal environments were determined using fixed classes defined jointly with the crop improvement team.

Table S5. WSPD at peak under each stress pattern in each country under present and future climates.

| **Country** | **TPEs^1^** | **Hist.** | **2030** |  |  |  | **2050** |  |  |  |
| --- | --- | --- | --- | --- | --- | --- | --- | --- | --- | --- |
|  |  |  | **SSP1-2.6** | **SSP2-4.5** | **SSP3-7.0** | **SSP5-8.5** | **SSP1-2.6** | **SSP2-4.5** | **SSP3-7.0** | **SSP5-8.5** |
| Uganda | SF | 0 | 0.02 | 0.02 | 0.02 | 0.02 | 0.02 | 0.02 | 0.02 | 0.3 |
|  | STS | 0.6 | 0.51 | 0.54 | 0.5 | 0.54 | 0.52 | 0.51 | 0.46 | 0.52 |
| Ethiopia | SF | 0 | 0.02 | 0.02 | 0.02 | 0.02 | 0.02 | 0.02 | 0.02 | 0.02 |
|  | MTS | 0.4 | 0.42 | 0.44 | 0.49 | 0.47 | 0.5 | 0.45 | 0.45 | 0.47 |
|  | ETS | 0.75 | 0.72 | 0.73 | 0.74 | 0.71 | 0.73 | 0.7 | 0.71 | 0.67 |
|  | GS | 0.43 | 0.5 | 0.49 | 0.48 | 0.47 | 0.47 | 0.46 | 0.44 | 0.42 |
| Tanzania | SF | 0 | 0.01 | 0 | 0.02 | 0.01 | 0.01 | 0.01 | 0.01 | 0.01 |
|  | AS | 0.46 | 0.25 | 0.25 | 0.22 | 0.2 | 0.15 | 0.48 | 0.41 | 0.3 |
|  | MTS | 0.41 | 0.48 | 0.34 | 0.49 | 0.44 | 0.42 | 0.38 | 0.44 | 0.4 |
|  | STS | 0.7 | 0.68 | 0.66 | 0.64 | 0.63 | 0.67 | 0.62 | 0.64 | 0.65 |
|  | ETS | 0.75 | 0.82 | 0.79 | 0.8 | 0.8 | 0.81 | 0.82 | 0.79 | 0.8 |

Table S6. Bean harvested area (in ‘000 hectares) under various target population of environmental groups in different production hubs based on the multi-model mean for each SSP.

| **Country** | **Production hub** | **TPE^1^** | **History** | **2030** | | | | **2050** | | | |
| --- | --- | --- | --- | --- | --- | --- | --- | --- | --- | --- | --- |
|  |  |  |  | **SSP**  **1-2.6** | **SSP**  **2-4.5** | **SSP**  **3-7.0** | **SSP**  **5-8.5** | **SSP**  **1-2.6** | **SSP**  **2-4.5** | **SSP**  **3-7.0** | **SSP**  **5-8.5** |
| UGA | Eastern Tall Grass | SF | 67.018 | 56.209 | 61.253 | 60.532 | 58.371 | 56.929 | 56.209 | 53.326 | 55.488 |
| UGA | Eastern Tall Grass | STS | 5.044 | 15.854 | 10.809 | 11.53 | 13.692 | 15.133 | 15.854 | 18.736 | 16.574 |
| UGA | Mt Elgon | SF | 30.632 | 29.654 | 30.632 | 30.306 | 30.306 | 29.654 | 29.654 | 28.677 | 29.003 |
| UGA | Mt Elgon | STS | 1.955 | 2.933 | 1.955 | 2.281 | 2.281 | 2.933 | 2.933 | 3.91 | 3.585 |
| UGA | North Central | SF | 77.367 | 71.606 | 74.075 | 73.252 | 74.075 | 70.783 | 71.606 | 67.491 | 69.137 |
| UGA | North Central | STS | 4.938 | 10.7 | 8.231 | 9.054 | 8.231 | 11.523 | 10.7 | 14.815 | 13.169 |
| UGA | Northern Short Grass | SF | 28.082 | 27.458 | 28.394 | 28.082 | 28.394 | 27.458 | 27.458 | 26.21 | 26.834 |
| UGA | Northern Short Grass | STS | 3.12 | 3.744 | 2.808 | 3.12 | 2.808 | 3.744 | 3.744 | 4.992 | 4.368 |
| UGA | South-western Highlands | SF | 17.085 | 8.937 | 10.251 | 9.988 | 11.04 | 9.2 | 9.463 | 9.2 | 9.725 |
| UGA | South-western Highlands | STS | 9.2 | 17.348 | 16.034 | 16.297 | 15.245 | 17.085 | 16.822 | 17.085 | 16.56 |
| UGA | South-western Tall Grass | SF | 42.476 | 23.059 | 26.699 | 26.093 | 28.52 | 23.665 | 23.665 | 23.059 | 23.665 |
| UGA | South-western Tall Grass | STS | 18.204 | 37.622 | 33.981 | 34.588 | 32.161 | 37.015 | 37.015 | 37.622 | 37.015 |
| UGA | Western Highlands | SF | 20.953 | 15.106 | 16.567 | 16.324 | 16.567 | 14.375 | 15.836 | 14.862 | 15.593 |
| UGA | Western Highlands | STS | 3.411 | 9.258 | 7.796 | 8.284 | 7.796 | 9.989 | 8.527 | 9.502 | 8.771 |
| UGA | Western Short Grass | SF | 74.187 | 46.632 | 51.931 | 50.871 | 54.051 | 46.632 | 48.752 | 45.572 | 47.692 |
| UGA | Western Short Grass | STS | 31.794 | 59.35 | 54.051 | 55.11 | 51.931 | 59.35 | 57.23 | 60.41 | 58.29 |
| UGA | Western Tall Grass | SF | 112.673 | 91.151 | 97.481 | 94.949 | 97.481 | 87.353 | 91.151 | 86.087 | 89.885 |
| UGA | Western Tall Grass | STS | 13.926 | 35.448 | 29.118 | 31.65 | 29.118 | 39.246 | 35.448 | 40.512 | 36.714 |
| UGA | North-western Tall Grass | SF | 15.725 | 14.387 | 14.889 | 14.554 | 14.889 | 14.052 | 14.554 | 13.55 | 14.22 |
| UGA | North-western Tall Grass | STS | 1.004 | 2.342 | 1.84 | 2.175 | 1.84 | 2.677 | 2.175 | 3.178 | 2.509 |
| ETH | Central Rift Valley | GS | 4.569 | 4.218 | 3.515 | 3.866 | 3.515 | 3.866 | 2.812 | 3.515 | 3.163 |
| ETH | Central Rift Valley | SF | 16.168 | 19.683 | 19.683 | 20.385 | 19.331 | 20.385 | 20.737 | 19.683 | 21.791 |
| ETH | Central Rift Valley | MTS | 10.544 | 5.975 | 7.381 | 7.029 | 7.381 | 6.678 | 5.975 | 7.381 | 5.975 |
| ETH | Central Rift Valley | ETS | 3.866 | 5.272 | 4.569 | 3.866 | 4.921 | 4.218 | 5.624 | 4.569 | 4.218 |
| ETH | Amhara region | GS | 0.192 | 0.23 | 0.23 | 0.23 | 0.23 | 0.192 | 0.153 | 0.23 | 0.153 |
| ETH | Amhara region | SF | 1.342 | 1.687 | 1.725 | 1.916 | 1.725 | 1.993 | 2.031 | 2.07 | 2.338 |
| ETH | Amhara region | MTS | 1.648 | 1.035 | 1.188 | 1.15 | 1.265 | 1.035 | 0.92 | 1.035 | 0.997 |
| ETH | Amhara region | ETS | 0.652 | 0.882 | 0.69 | 0.537 | 0.613 | 0.613 | 0.728 | 0.498 | 0.345 |
| ETH | Hararghe | GS | 5.633 | 4.116 | 4.116 | 3.466 | 3.25 | 3.9 | 3.466 | 3.683 | 2.816 |
| ETH | Hararghe | SF | 8.016 | 9.099 | 9.316 | 10.182 | 10.182 | 10.399 | 10.182 | 10.616 | 10.832 |
| ETH | Hararghe | MTS | 6.716 | 4.766 | 5.416 | 6.066 | 5.849 | 4.983 | 4.766 | 4.766 | 5.849 |
| ETH | Hararghe | ETS | 1.3 | 3.683 | 2.816 | 1.95 | 2.383 | 2.383 | 3.25 | 2.6 | 2.166 |
| ETH | North East | GS | 0.023 | 0.012 | 0.017 | 0.012 | 0.012 | 0.012 | 0.012 | 0.01 | 0.016 |
| ETH | North East | SF | 0.018 | 0.028 | 0.028 | 0.031 | 0.028 | 0.031 | 0.031 | 0.033 | 0.027 |
| ETH | North East | MTS | 0.03 | 0.021 | 0.022 | 0.026 | 0.025 | 0.023 | 0.02 | 0.021 | 0.023 |
| ETH | North East | ETS | 0.018 | 0.029 | 0.022 | 0.02 | 0.024 | 0.022 | 0.027 | 0.025 | 0.023 |
| ETH | North Shewa | GS | 0 | 0.008 | 0.015 | 0.015 | 0.012 | 0.012 | 0.012 | 0.012 | 0.012 |
| ETH | North Shewa | SF | 0.088 | 0.134 | 0.13 | 0.142 | 0.13 | 0.138 | 0.142 | 0.15 | 0.18 |
| ETH | North Shewa | MTS | 0.165 | 0.104 | 0.119 | 0.138 | 0.134 | 0.127 | 0.104 | 0.127 | 0.146 |
| ETH | North Shewa | ETS | 0.13 | 0.138 | 0.119 | 0.088 | 0.107 | 0.107 | 0.127 | 0.096 | 0.046 |
| ETH | Pawe | GS | 0.036 | 0.071 | 0.071 | 0.107 | 0.107 | 0.071 | 0.071 | 0.071 | 0.071 |
| ETH | Pawe | SF | 2.884 | 2.955 | 2.991 | 3.062 | 2.955 | 3.027 | 3.133 | 2.706 | 3.169 |
| ETH | Pawe | MTS | 0.605 | 0.356 | 0.392 | 0.249 | 0.392 | 0.356 | 0.285 | 0.677 | 0.249 |
| ETH | Pawe | ETS | 0.036 | 0.178 | 0.107 | 0.142 | 0.107 | 0.107 | 0.071 | 0.107 | 0.071 |
| ETH | Southern Rift Valley | GS | 9.573 | 8.296 | 9.573 | 8.935 | 7.658 | 9.573 | 8.296 | 7.658 | 5.744 |
| ETH | Southern Rift Valley | SF | 44.673 | 44.035 | 45.949 | 46.588 | 47.226 | 46.588 | 47.226 | 40.206 | 48.502 |
| ETH | Southern Rift Valley | MTS | 8.296 | 6.382 | 4.467 | 4.467 | 4.467 | 3.191 | 3.191 | 11.487 | 5.744 |
| ETH | Southern Rift Valley | ETS | 1.276 | 5.105 | 3.829 | 3.829 | 4.467 | 4.467 | 5.105 | 4.467 | 3.829 |
| ETH | Welkite and Silte | GS | 0.077 | 0.06 | 0.068 | 0.06 | 0.051 | 0.077 | 0.051 | 0.06 | 0.06 |
| ETH | Welkite and Silte | SF | 0.359 | 0.444 | 0.436 | 0.47 | 0.453 | 0.478 | 0.495 | 0.487 | 0.538 |
| ETH | Welkite and Silte | MTS | 0.333 | 0.205 | 0.248 | 0.222 | 0.248 | 0.214 | 0.196 | 0.205 | 0.179 |
| ETH | Welkite and Silte | ETS | 0.085 | 0.145 | 0.103 | 0.103 | 0.103 | 0.085 | 0.111 | 0.103 | 0.077 |
| ETH | West Wellega | GS | 0.139 | 0.278 | 0.278 | 0.417 | 0.417 | 0.417 | 0.278 | 0.278 | 0.417 |
| ETH | West Wellega | SF | 12.787 | 12.37 | 12.509 | 12.509 | 12.37 | 12.509 | 12.787 | 12.37 | 12.509 |
| ETH | West Wellega | MTS | 0.973 | 0.834 | 0.834 | 0.695 | 0.834 | 0.695 | 0.556 | 0.973 | 0.695 |
| ETH | West Wellega | ETS | 0 | 0.417 | 0.278 | 0.278 | 0.278 | 0.278 | 0.278 | 0.278 | 0.278 |
| ETH | Western | GS | 0 | 0.723 | 0.723 | 0.723 | 0.723 | 0.723 | 0.542 | 0.542 | 0.542 |
| ETH | Western | SF | 11.932 | 12.112 | 12.655 | 13.197 | 12.836 | 13.378 | 13.74 | 13.559 | 14.282 |
| ETH | Western | MTS | 5.423 | 3.435 | 3.616 | 3.254 | 3.435 | 2.893 | 2.712 | 2.893 | 2.531 |
| ETH | Western | ETS | 0.723 | 1.808 | 1.085 | 0.904 | 1.085 | 1.085 | 1.085 | 1.085 | 0.723 |
| TZA | Kagera | SF | 101.397 | 97.901 | 97.901 | 97.901 | 94.404 | 87.412 | 96.735 | 100.232 | 96.735 |
| TZA | Kagera | AS | 11.655 | 5.827 | 3.496 | 6.993 | 8.158 | 13.986 | 4.662 | 3.496 | 3.496 |
| TZA | Kagera | MTS | 1.165 | 8.158 | 10.489 | 6.993 | 9.324 | 10.489 | 10.489 | 8.158 | 9.324 |
| TZA | Kagera | STS | 1.165 | 3.496 | 3.496 | 3.496 | 3.496 | 3.496 | 3.496 | 3.496 | 4.662 |
| TZA | Kagera | ETS | 1.165 | 1.165 | 1.165 | 1.165 | 1.165 | 1.165 | 1.165 | 1.165 | 2.331 |
| TZA | Kigoma | SF | 75.577 | 75.577 | 74.556 | 79.663 | 76.599 | 75.577 | 71.492 | 77.62 | 75.577 |
| TZA | Kigoma | AS | 19.405 | 2.043 | 4.085 | 3.064 | 2.043 | 3.064 | 5.107 | 2.043 | 2.043 |
| TZA | Kigoma | MTS | 4.085 | 12.256 | 12.256 | 11.234 | 13.277 | 12.256 | 13.277 | 12.256 | 11.234 |
| TZA | Kigoma | STS | 1.021 | 7.149 | 8.171 | 5.107 | 7.149 | 8.171 | 8.171 | 6.128 | 9.192 |
| TZA | Kigoma | ETS | 2.043 | 5.107 | 3.064 | 3.064 | 3.064 | 3.064 | 4.085 | 4.085 | 4.085 |
| TZA | Shinyanga | SF | 20.259 | 22.236 | 19.765 | 23.718 | 20.754 | 20.754 | 21.248 | 22.236 | 21.248 |
| TZA | Shinyanga | AS | 17.789 | 2.965 | 3.953 | 2.965 | 2.965 | 2.965 | 4.447 | 1.482 | 1.977 |
| TZA | Shinyanga | MTS | 7.412 | 10.871 | 9.883 | 10.377 | 11.365 | 9.883 | 10.871 | 10.871 | 9.389 |
| TZA | Shinyanga | STS | 0.494 | 7.906 | 10.871 | 7.412 | 8.894 | 10.871 | 8.4 | 8.894 | 10.377 |
| TZA | Shinyanga | ETS | 3.459 | 5.435 | 4.941 | 4.941 | 5.435 | 4.941 | 4.447 | 5.93 | 6.424 |
| TZA | Maara | SF | 5.76 | 6.84 | 4.86 | 7.92 | 7.2 | 4.86 | 5.04 | 5.58 | 5.4 |
| TZA | Maara | AS | 8.46 | 1.44 | 1.62 | 0.72 | 0.54 | 0.9 | 1.8 | 1.08 | 2.34 |
| TZA | Maara | MTS | 1.44 | 5.58 | 5.76 | 5.58 | 6.48 | 5.76 | 6.3 | 6.3 | 4.5 |
| TZA | Maara | STS | 0.18 | 3.24 | 4.86 | 3.06 | 3.06 | 5.22 | 3.78 | 3.42 | 3.78 |
| TZA | Maara | ETS | 2.16 | 0.9 | 0.9 | 0.72 | 0.72 | 1.26 | 1.08 | 1.62 | 1.98 |
| TZA | Northern Highlands | SF | 7.568 | 6.487 | 6.847 | 6.667 | 5.586 | 5.586 | 6.847 | 7.387 | 6.487 |
| TZA | Northern Highlands | AS | 3.784 | 3.784 | 2.523 | 3.063 | 4.324 | 3.784 | 2.342 | 1.982 | 2.342 |
| TZA | Northern Highlands | MTS | 3.604 | 2.342 | 2.703 | 2.523 | 2.703 | 2.703 | 2.703 | 2.883 | 2.703 |
| TZA | Northern Highlands | STS | 1.982 | 2.703 | 3.243 | 3.063 | 3.063 | 3.063 | 3.063 | 2.703 | 3.243 |
| TZA | Northern Highlands | ETS | 1.081 | 2.703 | 2.703 | 2.703 | 2.342 | 2.883 | 3.063 | 3.063 | 3.243 |
| TZA | Northern Semi-arid Highlands | SF | 49.782 | 38.719 | 41.485 | 41.485 | 33.188 | 33.188 | 45.633 | 45.633 | 41.485 |
| TZA | Northern Semi-arid Highlands | AS | 30.422 | 30.422 | 17.977 | 22.125 | 29.039 | 27.657 | 17.977 | 15.211 | 16.594 |
| TZA | Northern Semi-arid Highlands | MTS | 33.188 | 17.977 | 20.742 | 19.36 | 20.742 | 22.125 | 19.36 | 20.742 | 19.36 |
| TZA | Northern Semi-arid Highlands | STS | 12.445 | 27.657 | 31.805 | 29.039 | 29.039 | 29.039 | 29.039 | 24.891 | 29.039 |
| TZA | Northern Semi-arid Highlands | ETS | 12.445 | 23.508 | 26.274 | 26.274 | 26.274 | 26.274 | 26.274 | 31.805 | 31.805 |
| TZA | Tanga | SF | 20.192 | 17.949 | 18.269 | 18.91 | 16.987 | 15.705 | 18.59 | 19.231 | 17.308 |
| TZA | Tanga | AS | 5.449 | 6.731 | 4.167 | 5.128 | 6.41 | 7.051 | 3.205 | 3.526 | 4.167 |
| TZA | Tanga | MTS | 1.603 | 3.846 | 5.128 | 3.846 | 4.167 | 4.808 | 5.449 | 4.808 | 5.449 |
| TZA | Tanga | STS | 3.526 | 2.244 | 3.205 | 2.885 | 3.205 | 2.885 | 3.205 | 2.885 | 3.205 |
| TZA | Tanga | ETS | 1.282 | 1.282 | 1.282 | 1.282 | 1.282 | 1.603 | 1.603 | 1.603 | 1.923 |
| TZA | Mpanda | SF | 4.726 | 4.849 | 4.604 | 4.91 | 4.788 | 4.726 | 4.665 | 4.849 | 4.788 |
| TZA | Mpanda | AS | 1.043 | 0.123 | 0.246 | 0.184 | 0.123 | 0.184 | 0.246 | 0.123 | 0.061 |
| TZA | Mpanda | MTS | 0.123 | 0.737 | 0.737 | 0.675 | 0.798 | 0.675 | 0.798 | 0.737 | 0.675 |
| TZA | Mpanda | STS | 0.061 | 0.307 | 0.491 | 0.246 | 0.307 | 0.43 | 0.307 | 0.307 | 0.491 |
| TZA | Mpanda | ETS | 0.184 | 0.123 | 0.061 | 0.123 | 0.123 | 0.123 | 0.123 | 0.123 | 0.123 |
| TZA | Southern Highlands | SF | 145.986 | 138.806 | 134.02 | 141.2 | 134.02 | 129.233 | 134.02 | 138.806 | 136.413 |
| TZA | Southern Highlands | AS | 38.291 | 14.359 | 14.359 | 16.752 | 14.359 | 16.752 | 16.752 | 9.573 | 7.18 |
| TZA | Southern Highlands | MTS | 35.898 | 28.719 | 28.719 | 26.325 | 31.112 | 28.719 | 28.719 | 31.112 | 26.325 |
| TZA | Southern Highlands | STS | 2.393 | 26.325 | 31.112 | 23.932 | 31.112 | 33.505 | 28.719 | 28.719 | 35.898 |
| TZA | Southern Highlands | ETS | 16.752 | 31.112 | 31.112 | 31.112 | 28.719 | 31.112 | 31.112 | 31.112 | 33.505 |
| TZA | Southern Mid-altitude | SF | 18.65 | 16.319 | 15.853 | 15.853 | 14.92 | 14.92 | 15.853 | 16.319 | 16.319 |
| TZA | Southern Mid-altitude | AS | 6.528 | 3.264 | 3.264 | 3.73 | 4.196 | 3.73 | 3.264 | 2.798 | 1.865 |
| TZA | Southern Mid-altitude | MTS | 17.718 | 4.663 | 4.196 | 4.663 | 5.129 | 4.663 | 4.663 | 5.129 | 4.663 |
| TZA | Southern Mid-altitude | STS | 0.466 | 9.325 | 9.325 | 7.926 | 7.926 | 10.724 | 9.325 | 6.994 | 9.791 |
| TZA | Southern Mid-altitude | ETS | 3.264 | 13.055 | 13.988 | 14.454 | 14.454 | 12.589 | 13.521 | 15.387 | 13.988 |
| TZA | Southern Lowlands | SF | 12.306 | 11.152 | 10.767 | 11.344 | 10.575 | 10.383 | 10.96 | 11.344 | 11.344 |
| TZA | Southern Lowlands | AS | 3.076 | 1.538 | 1.346 | 1.73 | 1.538 | 1.73 | 1.346 | 0.961 | 0.769 |
| TZA | Southern Lowlands | MTS | 2.307 | 2.115 | 2.5 | 2.115 | 2.692 | 2.307 | 2.5 | 2.5 | 2.115 |
| TZA | Southern Lowlands | STS | 0.192 | 2.307 | 2.692 | 1.923 | 2.307 | 2.692 | 2.307 | 2.115 | 2.5 |
| TZA | Southern Lowlands | ETS | 1.346 | 2.115 | 1.923 | 2.115 | 2.115 | 2.115 | 2.115 | 2.307 | 2.5 |

^1^ Target Population of Environments (TPEs) are as follows: SF= stress free; AS = all-season stress; MTS = moderate terminal stress; STS = severe terminal stress; ETS = extreme terminal stress; GS = grain filling stress.

Table S7. Area (in 000 ha) under each TPE in each country in each temperature class under historical and future climates.

| Country | T_min_ | TPE | Historical | 2030 | | 2050 | |
| --- | --- | --- | --- | --- | --- | --- | --- |
|  |  |  |  | SSP1-2.6 | SSP5-5.8 | SSP1-2.6 | SSP5-5.8 |
| Ethiopia | <15°C | SF | 82.596 | 66.011 | 21.667 | 23.025 | 21.818 |
|  | 15-18°C | SF | 15.671 | 24.166 | 73.199 | 73.392 | 76.672 |
|  | 18-20°C | SF | 0 | 12.37 | 12.37 | 12.509 | 15.678 |
|  | 20-22ºC | SF | 0 | 0 | 0 | 0 | 0 |
|  | >22ºC | SF | 0 | 0 | 0 | 0 | 0 |
|  | <15°C | GS | 20.067 | 12.824 | 3.82 | 4.159 | 3.179 |
|  | 15-18°C | GS | 0.175 | 4.91 | 11.738 | 14.267 | 9.327 |
|  | 18-20°C | GS | 0 | 0.278 | 0.417 | 0.417 | 0.488 |
|  | 20-22ºC | GS | 0 | 0 | 0 | 0 | 0 |
|  | >22ºC | GS | 0 | 0 | 0 | 0 | 0 |
|  | <15°C | MTS | 33.155 | 13.722 | 9.053 | 8.077 | 5.998 |
|  | 15-18°C | MTS | 1.578 | 8.557 | 14.143 | 11.423 | 15.446 |
|  | 18-20°C | MTS | 0 | 0.834 | 0.834 | 0.695 | 0.944 |
|  | 20-22ºC | MTS | 0 | 0 | 0 | 0 | 0 |
|  | >22ºC | MTS | 0 | 0 | 0 | 0 | 0 |
|  | <15°C | ETS | 8.05 | 11.571 | 5.768 | 5.045 | 4.241 |
|  | 15-18°C | ETS | 0.036 | 5.669 | 8.042 | 8.042 | 7.186 |
|  | 18-20°C | ETS | 0 | 0.417 | 0.278 | 0.278 | 0.349 |
|  | 20-22ºC | ETS | 0 | 0 | 0 | 0 | 0 |
|  | >22ºC | ETS | 0 | 0 | 0 | 0 | 0 |
| Tanzania | <15°C | SF | 153.554 | 6.487 | 0 | 0 | 0 |
|  | 15-18°C | SF | 288.39 | 396.96 | 372.772 | 351.561 | 160.208 |
|  | 18-20°C | SF | 20.259 | 33.388 | 46.249 | 50.783 | 251.648 |
|  | 20-22°C | SF | 0 | 0 | 0 | 0 | 21.248 |
|  | >22ºC | SF | 0 | 0 | 0 | 0 | 0 |
|  | <15°C | AS | 42.075 | 3.784 | 0 | 0 | 0 |
|  | 15-18°C | AS | 86.038 | 64.209 | 64.996 | 73.194 | 13.689 |
|  | 18-20°C | AS | 17.789 | 4.503 | 9.699 | 8.609 | 27.168 |
|  | 20-22°C | AS | 0 | 0 | 0 | 0 | 1.977 |
|  | >22ºC | AS | 0 | 0 | 0 | 0 | 0 |
|  | <15°C | MTS | 39.502 | 2.342 | 0 | 0 | 0 |
|  | 15-18°C | MTS | 61.629 | 81.936 | 88.603 | 86.86 | 34.477 |
|  | 18-20°C | MTS | 7.412 | 12.986 | 19.186 | 17.528 | 51.871 |
|  | 20-22°C | MTS | 0 | 0 | 0 | 0 | 9.389 |
|  | >22ºC | MTS | 0 | 0 | 0 | 0 | 0 |
|  | <15°C | STS | 4.375 | 2.703 | 0 | 0 | 0 |
|  | 15-18°C | STS | 19.056 | 79.743 | 80.431 | 85.379 | 42.346 |
|  | 18-20°C | STS | 0.494 | 10.213 | 19.127 | 24.717 | 59.455 |
|  | 20-22°C | STS | 0 | 0 | 0 | 0 | 10.377 |
|  | >22ºC | STS | 0 | 0 | 0 | 0 | 0 |
|  | <15°C | ETS | 17.833 | 2.703 | 0 | 0 | 0 |
|  | 15-18°C | ETS | 23.889 | 76.252 | 63.689 | 67.361 | 38.671 |
|  | 18-20°C | ETS | 3.459 | 7.55 | 22.004 | 19.768 | 56.812 |
|  | 20-22°C | ETS | 0 | 0 | 0 | 0 | 6.424 |
|  | >22ºC | ETS | 0 | 0 | 0 | 0 | 0 |
| Uganda | <15°C | SF | 90.193 | 8.937 | 11.04 | 0 | 0 |
|  | 15-18°C | SF | 274.831 | 114.451 | 112.877 | 109.151 | 62.393 |
|  | 18-20°C | SF | 105.449 | 246.424 | 274.888 | 186.115 | 208.658 |
|  | 20-22°C | SF | 15.725 | 14.387 | 14.889 | 84.835 | 95.971 |
|  | >22°C | SF | 0 | 0 | 0 | 0 | 14.22 |
|  | <15°C | STS | 29.359 | 17.348 | 15.245 | 0 | 0 |
|  | 15-18°C | STS | 54.175 | 109.163 | 86.373 | 116.383 | 57.16 |
|  | 18-20°C | STS | 8.058 | 65.746 | 61.645 | 68.112 | 120.349 |
|  | 20-22°C | STS | 1.004 | 2.342 | 1.84 | 14.2 | 17.537 |
|  | >22°C | STS | 0 | 0 | 0 | 0 | 2.509 |

^1^ Target Population of Environments (TPEs) are as follows: SF= stress free; AS = all-season stress; MTS = moderate terminal stress; STS = severe terminal stress; ETS = extreme terminal stress; GS = grain filling stress; UGA= Uganda; ETH=Ethiopia; TZA=Tanzania

1. Corresponding author.

   *Email address*: [jhaprak@gmail.com](mailto:jhaprak@gmail.com) (P.K. Jha). [↑](#footnote-ref-1)
2. The model is available as an R package at <https://github.com/CIAT-DAPA/analogues> [↑](#footnote-ref-2)
